# PTPN2 Inhibition Disrupts Mitochondrial Renewal and Blocks TFRC‐Mediated Mitophagy to Exert Anti‐Tumor Activities in ALK‐Positive Anaplastic Large Cell Lymphoma

**DOI:** 10.1002/advs.202414282

**Published:** 2025-07-30

**Authors:** Wei‐Ting Wang, Zi‐Wen Duan, Tong‐Yao Xing, Wei Hua, Kai‐Xing Du, Chun‐Yu Shang, Yi‐Fan Wu, Li Wang, Jian‐Yong Li, Rui Gao, Jin‐Hua Liang, Wei Xu

**Affiliations:** ^1^ Department of Hematology The First Affiliated Hospital with Nanjing Medical University Jiangsu Province Hospital Nanjing 210029 China; ^2^ Key Laboratory of Hematology of Nanjing Medical University Nanjing 210029 China; ^3^ Collaborative Innovation Center for Cancer Personalized Medicine Nanjing 210029 China; ^4^ Department of Endocrinology The First Affiliated Hospital with Nanjing Medical University Jiangsu Province Hospital Nanjing 210029 China

**Keywords:** ABBV‐CLS‐484, ALK‐positive anaplastic large cell lymphoma, mitophagy, PTPN2, TFRC

## Abstract

Anaplastic large cell lymphoma (ALCL) is a heterogeneous subtype of T‐cell lymphoma usually driven by genetic alterations affecting the anaplastic lymphoma kinase (ALK) gene. Despite the relatively favorable prognosis of ALK‐positive (ALK^+^) ALCL, approximately 30–40% of patients experience relapses or disease progression. This work identifies protein tyrosine phosphatase PTPN2 as a critical gene essential for the growth and survival of ALK^+^ ALCL by CRISPR/Cas9 editing. PTPN2 depletion can significantly suppress tumor cell proliferation, induce apoptosis, and provoke cell cycle arrest. Mechanistically, PTPN2 negatively regulates transferrin receptor (TFRC) expression to promote mitochondrial renewal via PTEN induced kinase 1 (PINK1)‐PRKN (parkin RBR E3 ubiquitin protein ligase)‐mediated mitophagy. The process functions independently of ferroptosis. Interestingly, TFRC is directly regulated by the transcription factor hypoxia‐inducible factor 1 alpha (HIF1A) in its promoter. Notably, an orally bioavailable potent PTPN2/N1 active‐site inhibitor ABBV‐CLS‐484 (AC484) demonstrates significant therapeutic potential against ALK^+^ ALCL by disturbing mitochondrial renewal and blocking TFRC‐mediated PINK1‐PRKN‐dependent mitophagy to exert anti‐tumor activities, providing critical insights into the selection of targeted treatment strategies for ALK^+^ ALCL patients and a strong rationale for advancing AC484 into clinical trials.

## Graphical abstract

CRISPR/Cas9 screening reveals PTPN2 as essential for ALK^+^ ALCL survival. PTPN2 regulates TFRC to promote PINK1‐PRKN mitophagy. Oral PTPN2 inhibitor ABBV‐CLS‐484 disrupts mitochondrial renewal and blocks TFRC‐mediated mitophagy to exert anti‐tumor activities, supporting clinical development for ALK^+^ ALCL.

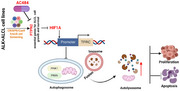

## 1 Introduction

Anaplastic large cell lymphoma (ALCL) is a heterogeneous subtype of T‐cell lymphoma predominantly driven by genetic alterations affecting the anaplastic lymphoma kinase (ALK) gene.^[^ ^1^ ^]^ The current standard treatments involve chemotherapy alone or in combination with brentuximab vedotin (BV), achieving a cure rate of approximately 70% in patients with ALK‐positive (ALK^+^) ALCL.^[^ ^2^ ^]^ Despite the relatively favorable prognosis of ALK^+^ ALCL, up to 30–40% of patients experience relapses or disease progression. Recommended treatment options for these patients include chemotherapy, clinical trials, BV, and ALK inhibitor crizotinib.^[^ ^3^ ^]^ However, just half of patients achieve complete remission (CR), and discontinuation of crizotinib usually leads to rapid relapse.^[^ ^4^ ^]^ As crizotinib cannot cover ALK‐mutated or resistant patients and the resistance mechanisms of BV remain unclear, exploring and developing chemotherapy‐free treatment strategies are critically necessary.

Protein tyrosine phosphatase PTPN2 (also called TCPTP) is a member of the largest class I cysteine protein tyrosine phosphatases family. PTPN2 functions by dephosphorylating target substrates like epidermal growth factor receptor (EGFR), Janus kinase (JAK), Src, and signal transducer and activator of transcription 3 (STAT3) to be involved in the inflammatory reaction, tumorigenesis, and immune regulation.^[^ ^5^ ^]^ Accumulating evidence highlights the tumor‐promoting effects of PTPN2 in breast cancer,^[^ ^6^ ^]^ hepatocellular carcinoma,^[^ ^7^ ^]^ and B‐cell lymphomas^[^ ^8^ ^]^ whereas PTPN2 is conversely involved in suppressing tumor growth in skin tumors.^[^ ^9^ ^]^ Recent in vivo CRISPR screening studies have identified PTPN2 as a cancer immunotherapy target.^[^ ^5^, ^10^ ^]^ Additionally, a first‐in‐class, orally bioavailable, potent PTPN2/N1 active‐site inhibitor ABBV‐CLS‐484 (AC484) has demonstrated promising therapeutic potential in solid tumors (NCT04777994).^[^ ^11^ ^]^ Despite these advances, the role of PTPN2 and the therapeutic application of AC484 in ALK^+^ ALCL remain unexplored.

Transferrin receptor (TFRC) plays a critical role in cellular iron transportation to maintain intracellular iron homeostasis.^[^ ^12^ ^]^ Intriguingly, Senyilmaz et al.^[^ ^13^ ^]^ have demonstrated that TFRC could induce mitochondrial fragmentation as a mitochondrial regulator. Mitophagy, an organelle‐specific form of autophagy, is responsible for the degradation of damaged or dysfunctional mitochondria to keep mitochondrial homeostasis.^[^ ^14^ ^]^ Recently, accumulating studies have proved the multifaceted role of mitophagy in tumorigenesis and tumor progression.^[^ ^15^ ^]^

Therefore, this study aims to investigate the role of PTPN2 and the efficacy of AC484 treatment in ALK^+^ ALCL. We demonstrate that PTPN2 exerts oncogenic effects by TFRC‐mediated mitophagy, independent of ferroptosis. Furthermore, AC484 exhibits strong potent anti‐tumor activity against ALK^+^ ALCL by disrupting mitochondrial function and inhibiting mitophagy. These findings provide critical insights into potential treatment strategies for ALK^+^ ALCL patients with poor prognosis and establish a strong rationale for advancing AC484 into clinical trials.

## 2 Results

### 2.1 PTPN2 is an Essential Oncogene with Elevated Expression in ALK^+^ ALCL

**Figure 1:**
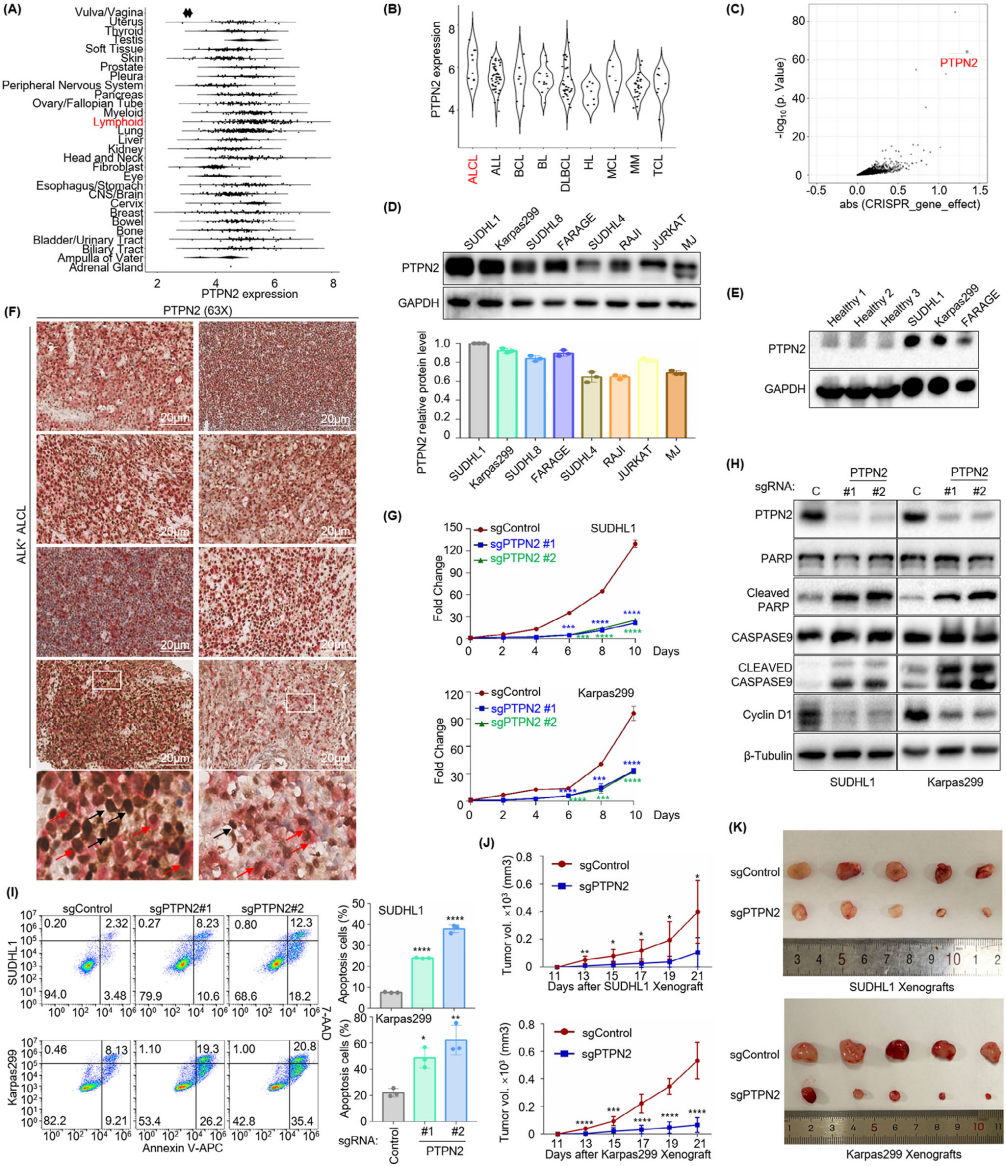
PTPN2 is an essential oncogene with elevated expression in ALK^+^ ALCL. A) Violin plots showing PTPN2 expression in all different types of tumor cell lines from DepMap. B) Violin plots showing PTPN2 expression in anaplastic large cell lymphoma (ALCL), acute lymphoblastic leukemia (ALL), B‐cell lymphoma (BCL), Burkitt lymphoma (BL), diffuse large B‐cell lymphoma (DLBCL), Hodgkin's lymphoma (HL), mantle cell lymphoma (MCL), multiple myeloma (MM), and T‐cell lymphoma (TCL) cell lines from DepMap. C) Scatter plots showing the gene effect scores of PTPN2 in 5 ALK^+^ ALCL models (SUDHL1, Karpas299, KIJK, SUPM2, and DEL) compared to other 1,081 cancer cell lines using the limma analysis method in the latest edition of the DepMap CRISPR screening datasets. D) Immunoblot analysis showing PTPN2 expression in ALK^+^ ALCL (SUDHL1, Karpas299), DLBCL (SUDHL8, FARAGE, SUDHL4), BL (RAJI), and T‐cell leukemia/lymphoma (JURKAT, MJ) cell lines. *n* = 3. E) Immunoblot analysis showing PTPN2 expression in lymphoma cell lines compared to CD3‐positive peripheral blood mononuclear cells from healthy human donors. *n* = 3. F) Immunohistochemistry staining assay showing ALK and PTPN2 expression in lymph node tissues of 8 ALK^+^ ALCL patients (The red arrows represented ALK/PTPN2 double‐positive; the black arrows represented ALK‐positive). Scale bar: 20 µm. *n* = 8. G) Growth curve analysis showing the proliferation rate of Cas9+ SUDHL1 (top) and Karpas299 (bottom) following control or PTPN2 targeting sgRNAs expression for 10 days. *n* = 3. H) Immunoblot analysis showing the expression of PTPN2, PARP, cleaved PARP, caspase9, cleaved caspase9, and cyclin D1 in Cas9+ SUDHL1 and Karpas299 following control (C) or PTPN2 targeting sgRNAs expression. I) Flow cytometry analysis showing cell apoptosis in Cas9+ SUDHL1 (top) and Karpas299 (bottom) that express control or PTPN2 sgRNAs using annexin V‐APC/7‐AAD Kit. *n* = 3. J, K) Growth curve (J) and volume analysis (K) showing tumor proliferation and size in xenograft mouse models constructed using Cas9+ SUDHL1 (top) and Karpas299 (bottom) following control or PTPN2 targeting sgRNAs expression. *n* = 5 per group. The data are shown as the mean ± SDs. *, *p* < 0.05; **, *p* < 0.01; ***, *p* < 0.001; ****, *p* < 0.0001. Statistical analysis in panels D, G, I, and J was performed by one‐way ANOVA with multiple comparisons.

To systemically identify potential targets for ALK^+^ ALCL, we analyzed the Cancer Dependency Map (DepMap, https://depmap.org/portal/), which contains the essential genes for cancer cell survival and molecular characterization of over 1000 cell lines by conducting genome‐wide CRISPR and shRNA screens and integrating data from multiple sources including Cancer Cell Line Encyclopedia (CCLE).^[^ ^16^ ^]^ By leveraging DepMap's extensive datasets and analytical tools, five ALCL cell lines (SUDHL1, Karpas299, KIJK, SUPM2, and DEL) were characterized, all of which demonstrated ALK positivity. The top 10 preferentially essential genes in five ALK^+^ ALCL cell lines were described in Table S1 (Supporting Information. Gene effects of ALK^+^ ALCL cell lines from Depmap). Notably, PTPN2 was consistently observed in the above ALK^+^ ALCL cell lines, which suggests its critical role in the pathogenesis of ALK^+^ ALCL. Additionally, PTPN2 exhibited ubiquitous and elevated expression in all types of tumor cell lines from the CCLE data (**Figure** **1**A,B, Supporting Information. PTPN2 Expression Public 24Q4). Further investigation into ALK^+^ ALCL models demonstrated that PTPN2 exhibited significantly lower gene effect scores (indicating a higher degree of dependency) compared to other cancer cell lines, suggesting its distinct oncogenic role in ALK^+^ ALCL (Figure 1C, Supporting Information. PTPN2 CRISPR [DepMap Public 24Q4 + Score Chronos]).

For further validation, PTPN2 expression was detected in B‐ and T‐cell lymphoma cell lines based on the CCLE Supporting Information. PTPN2 Expression Public 24Q4). A moderately higher PTPN2 expression was observed in ALK^+^ ALCL cell lines (SUDHL1 and Karpas299) compared to diffuse large B‐cell lymphoma (DLBCL) cell lines (SUDHL8, FARAGE, and SUDHL4), Burkitt lymphoma (BL) cell line RAJI, and T‐cell leukemia/lymphoma cell lines (JURKAT and MJ) (Figure 1D). Since FARAGE exhibits high PTPN2 expression but lacks negative gene effects Supporting Information. PTPN2 CRISPR [DepMap Public 24Q4 + Score Chronos]), we selected it as a negative control. Additionally, PTPN2 expression was significantly elevated in ALK^+^ ALCL cell lines compared to CD3‐positive peripheral blood mononuclear cells (CD3^+^ PBMCs) from healthy donors (*n* = 3) (Figure 1E). Consistent with these results, immunohistochemistry (IHC) staining assay revealed elevated PTPN2 expression in ALK‐positive tumor cells from the lymph node tissues of 8 ALK^+^ ALCL patients (Figure 1F), with detailed clinical characteristics summarized in Table S2 (Supporting Information).

Next, we investigated the role of PTPN2 in tumor growth and survival by introducing control or PTPN2‐targeting sgRNAs into ALK^+^ ALCL cell lines using CRISPR/Cas9 technique. Growth curve analysis revealed that PTPN2 silencing significantly decreased cell proliferation (Figure 1G). Additionally, PTPN2 knockout upregulated the expression of apoptosis‐related proteins (cleaved PARP and caspase9) and the cell cycle‐associated protein cyclin D1 (Figure 1H), which was further corroborated by flow cytometry analysis of apoptosis (Figure 1I). To validate these findings in vivo, we established ALK^+^ ALCL mouse xenograft models (*n* = 5 per group). Mice in the PTPN2 knockout group displayed a significantly reduced tumor growth rate (Figure 1J) and tumor size (Figure 1K). PTPN2 expression was also confirmed in subcutaneous tumors from the xenograft models (Figure S1A, Supporting Information). To assess the specificity of PTPN2's oncogenic role, we extended our analysis to other B‐ and T‐cell lymphoma cell lines. In contrast to ALK^+^ ALCL, PTPN2 knockout did not significantly impair cell proliferation, induce apoptosis, or exhibit tumor‐promoting effects in FARAGE, RAJI, and JURKAT (Figure S1B–F, Supporting Information). These results were consistent with the genome‐wide CRISPR and shRNA screens data from DepMap Supporting Information. PTPN2 CRISPR [DepMap Public 24Q4 + Score Chronos]). Collectively, we demonstrated that PTPN2 was upregulated and played a critical role in promoting tumor growth and survival specifically in ALK^+^ ALCL.

### 2.2 PTPN2 Promotes Mitochondrial Renewal via Mitophagy in ALK^+^ ALCL

**Figure 2:**
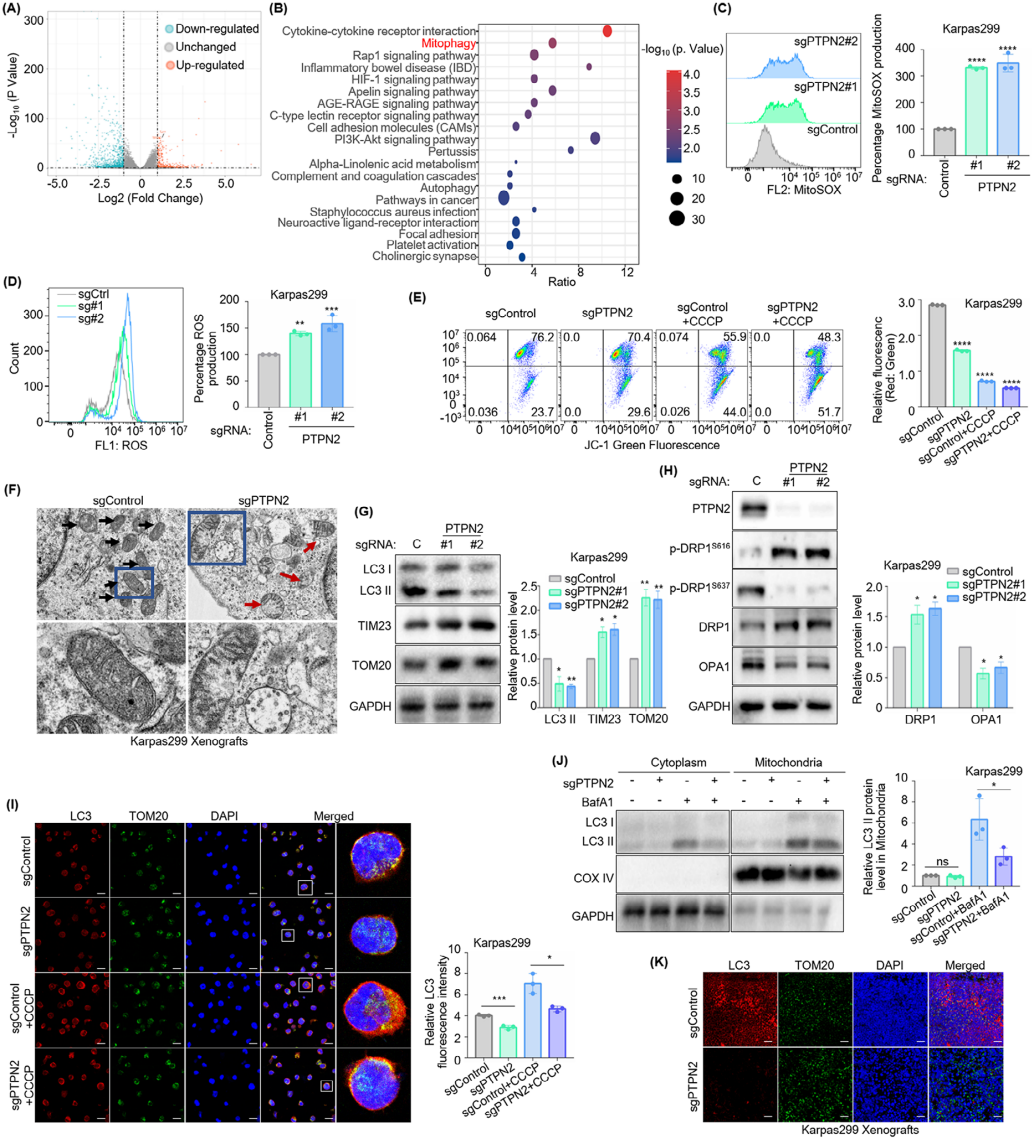
PTPN2 promotes mitochondrial renewal via mitophagy in ALK^+^ ALCL. A) Volcano plot showing down‐regulated and up‐regulated gene sets enriched in Cas9+ Karpas299 that express control (*n* = 3) or PTPN2 sgRNAs (*n* = 6) by RNA sequencing (RNA‐Seq) (*p* < 0.05, fold change > 1). B) Gene Set Enrichment Analysis of pathways showing the enrichment in ‘Mitophagy’ in Cas9+ Karpas299 that express control or PTPN2 sgRNAs by RNA‐Seq. C) MitoSOX analysis in Cas9+ Karpas299 that express control or PTPN2 sgRNAs. *n* = 3. D) ROS analysis in Cas9+ Karpas299 that express control (ctrl) or PTPN2 sgRNAs. *n* = 3. E) Mitochondrial membrane potential analysis in Cas9+ Karpas299 that express control or PTPN2 sgRNAs without or with 10 µ CCCP for 24 h by JC‐1. *n* = 3. F) Transmission electron microscopy analysis showing mitochondrial morphology in xenograft mouse models constructed using Cas9+ Karpas299 following control or PTPN2 targeting sgRNAs expression. x10k. *n* = 5. G) Immunoblot analysis showing the expression of LC3 I/II, TIM23, and TOM20 in Cas9+ Karpas299 that express control (C) or PTPN2 sgRNAs. *n* = 3. H) Immunoblot analysis showing the expression of p‐DRP1^S616^, p‐DRP1^S637^, DRP1, and OPA1 in Cas9+ Karpas299 that express control or PTPN2 sgRNAs. *n* = 3. I) Immunofluorescence analysis showing LC3 expression around mitochondria in Cas9+ Karpas299 that express control or PTPN2 sgRNAs without or with 10 µm CCCP for 24 h. Scale bar: 20 µm. *n* = 3. J) Immunoblot analysis showing LC3 I/II expression in the mitochondria of Cas9+ Karpas299 that express control or PTPN2 sgRNAs without or with 20 nm Bafilomycin A1 (BafA1) for 24 h. *n* = 3. K) Immunofluorescence analysis showing LC3 expression around mitochondria in xenograft mouse models constructed using Cas9+ Karpas299 following control or PTPN2 targeting sgRNAs expression. Scale bar: 20 µm. *n* = 5. The data are shown as the mean ± SDs. *, *p* < 0.05; **, *p* < 0.01; ***, *p* < 0.001; ****, *p* < 0.0001. Statistical analysis in panels C‐E, G, and H‐J was performed by one‐way ANOVA with multiple comparisons.

To investigate the potential molecular mechanism of PTPN2 in ALK^+^ ALCL, RNA sequencing (RNA‐seq) was performed on ALK^+^ ALCL cell line Karpas299 following PTPN2 knockout. Differential gene expression analysis (DEGs) identified a subset of genes significantly dysregulated upon PTPN2 depletion (*p* < 0.05, fold change > 1) (**Figure** **2**A). Subsequent KEGG pathway enrichment analysis revealed pronounced enrichment in the “Mitophagy” pathway, with key mitophagy‐related genes (HIF1A, JUN, RRAS, MITF, PRKN, MRAS, MAP1LC3C, MAP1LC3B2, SQSTM1, MAP1LC3B, TOMM20, OPA1, MFN1, TOMM7, and MFN2) exhibiting coordinated expression changes (Figure 2B). These findings implicated PTPN2 in regulating the mitophagy pathway, offering a mechanistic foundation for further investigation into its role in mitochondrial homeostasis. Growing studies have reported the involvement of mitophagy in lymphomagenesis.^[^ ^15^, ^17^ ^]^ To ascertain the role of PTPN2 on mitophagy, further experiments were performed. Flow cytometric analysis showed that the generation of mitochondrial superoxide (mitoSOX) (Figure 2C; Figure S2A, Supporting Information) and reactive oxygen species (ROS) (Figure 2D; Figure S2B, Supporting Information) was increased, while the mitochondrial membrane potential (MMP) (Figure 2E; Figure S2C, Supporting Information) were decreased following PTPN2 knockout. For further validation, the protonophore CCCP (carbonyl cyanide 3‐chlorophenylhydrazone) was introduced to induce mitochondrial damage. PTPN2 inhibition exacerbated the accumulation of damaged mitochondria upon CCCP treatment (Figure 2E; Figure S2C, Supporting Information). Additionally, transmission electron microscopy (TEM) analysis of mitochondria in subcutaneous tumors from ALK^+^ ALCL mouse xenograft models revealed that PTPN2‐deficient tumor cells exhibited an accumulation of swollen and damaged mitochondria (Figure 2F; Figure S2D, Supporting Information). Taken together, these results demonstrated that PTPN2 depletion induced mitochondrial damage and impaired mitochondrial function in ALK^+^ ALCL.

Increased mitochondrial fragmentation promotes mitophagy to facilitate mitochondrial renewal and eliminate damaged mitochondria.^[^ ^18^ ^]^ However, reduced expression of the autophagosome marker LC3 II was observed after PTPN2 knockout, accompanied by elevated expression of mitochondrial markers including TIM23 and TOM20 (Figure 2G; Figure S2E, Supporting Information), but not in the negative control FARAGE (Figure S2F, Supporting Information). Moreover, mitochondrial fission regulator dynamin‐related protein 1 (DRP1) and p‐DRP1^S616^ were upregulated while p‐DRP1^S637^ and the mitochondrial fusion‐related protein optic atrophy 1 (OPA1) were downregulated following PTPN2 knockout, indicating enhanced mitochondrial division and impaired mitochondrial fusion (Figure 2H; Figure S2G, Supporting Information).^[^ ^19^ ^]^ Immunofluorescence (IF) assay also detected reduced LC3 localization in mitochondria, as CCCP could induce the aggregation of LC3 around the mitochondria (Figure 2I; Figure S2H, Supporting Information). Additionally, LC3 II expression in mitochondria was significantly downregulated following PTPN2 knockout in the presence of Bafilomycin A1 (BafA1), as BafA1 could block autophagosome degradation (Figure 2J; Figure S2I, Supporting Information). Consistent with these findings, LC3 mitochondrial localization was diminished in subcutaneous tumors from ALK^+^ ALCL mouse xenograft models with PTPN2 knockout (Figure 2K; Figure S2J, Supporting Information). Overall, these results demonstrated that PTPN2 silencing led to mitochondrial dysfunction and inhibited mitophagy at early stages of development.

### 2.3 PTPN2 Suppresses TFRC Expression via HIF1A in ALK^+^ ALCL

**Figure 3:**
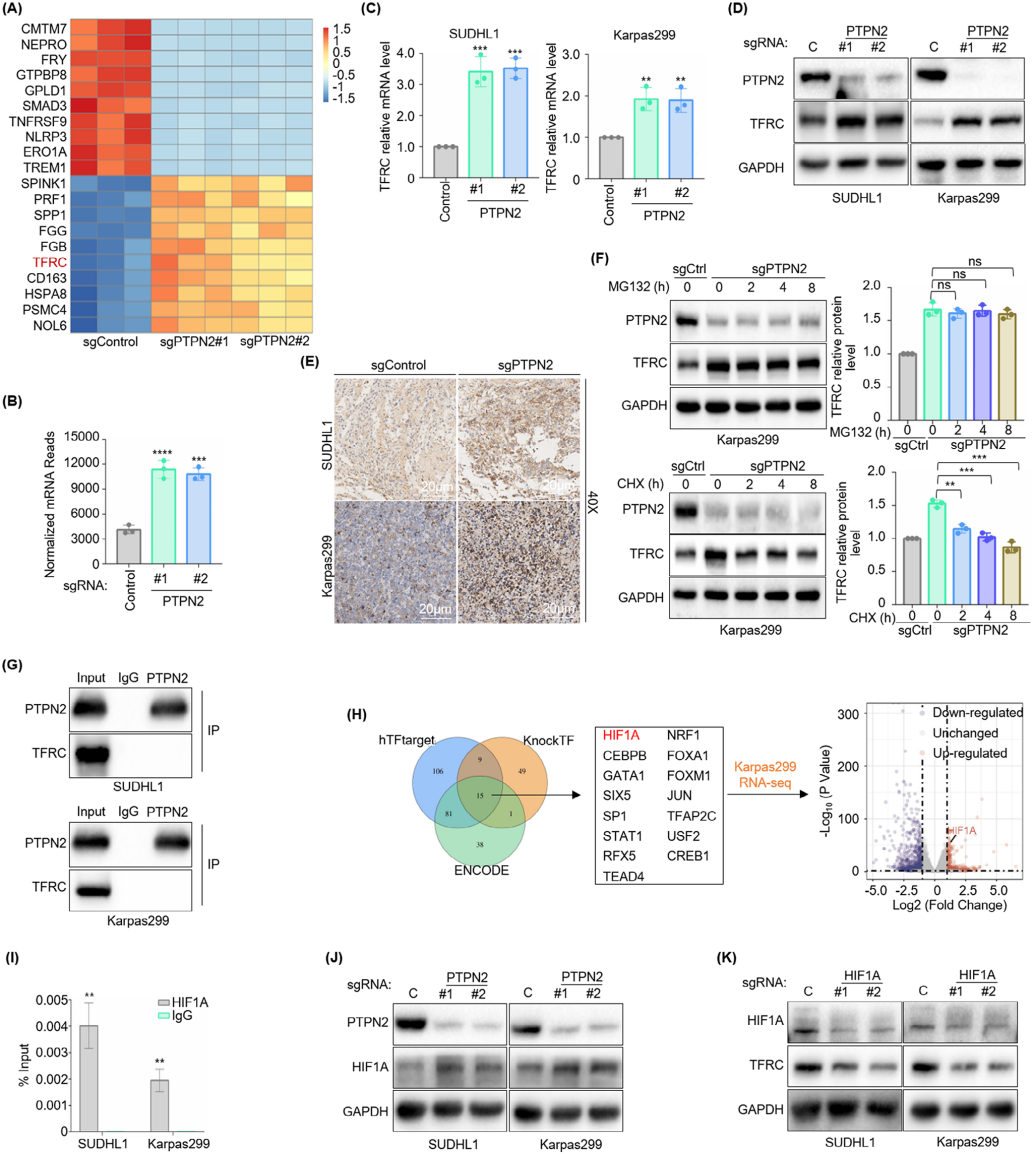
PTPN2 suppresses TFRC expression via HIF1A in ALK^+^ ALCL. A) Unsupervised hierarchical clustering heatmap showing the top 10 upregulated and top 10 downregulated genes enriched in Cas9+ Karpas299 that express control (*n* = 3) or PTPN2 sgRNAs (*n* = 6) by RNA‐Seq. B) Reads number analysis showing TFRC expression in Cas9+ Karpas299 that express control (*n* = 3) or PTPN2 sgRNAs (*n* = 6) by RNA‐Seq. C, D) RT‐qPCR (C) and immunoblot analysis (D) showing TFRC expression in Cas9+ SUDHL1 and Karpas299 that express control (C) or PTPN2 sgRNAs. *n* = 3. E) Immunohistochemistry staining assay showing TFRC expression in xenograft mouse models constructed using Cas9+ SUDHL1 (top) and Karpas299 (bottom) following control or PTPN2 targeting sgRNAs expression. Scale bar: 20 µm. *n* = 5. F) Immunoblot analysis showing TFRC expression in Cas9+ Karpas299 that express control or PTPN2 sgRNAs with 10 µm MG132 (top) or 1 µm cycloheximide (CHX) (bottom) for 0, 2, 4, and 8 h, respectively. *n* = 3. G) Co‐IP analysis showing no binding of PTPN2 and TFRC protein in SUDHL1 (top) and Karpas299 (bottom). H) Prediction analysis identifying HIF1A as the potential transcription factor of TFRC from prediction databases (hTFtarget, KnockTF, and ENCODE) combined with RNA‐Seq in Cas9+ Karpas299 that express control or PTPN2 sgRNAs. I) ChIP‐qPCR assay showing the binding of HIF1A and TFRC promoter (−2000 bp to −1 bp) in SUDHL1 and Karpas299 cells. *n* = 3. J) Immunoblot analysis showing HIF1A expression in Cas9+ SUDHL1 (left) and Karpas299 (right) that express control or PTPN2 sgRNAs. K) Immunoblot analysis showing TFRC expression in Cas9+ SUDHL1 (left) and Karpas299 (right) that express control or HIF1A sgRNAs. The data are shown as the mean ± SDs. *, *p* < 0.05; **, *p* < 0.01; ***, *p* < 0.001; ****, *p* < 0.0001. Statistical analysis in panels B, C, F, and I was performed by one‐way ANOVA with multiple comparisons.

The above results suggested that mitophagy might play tumor‐promoting effects on ALK^+^ ALCL. To further explore the potential effects of mitophagy on ALK^+^ ALCL, DEGs analysis was conducted. Notably, TFRC expression was markedly upregulated in PTPN2‐deficient ALK^+^ ALCL cells (Figures 2A and **3**A,B). Previous studies by Senyilmaz D. et al. have identified TFRC as a mitochondrial regulator capable of inducing mitochondrial fragmentation.^[^ ^13^ ^]^ Based on these observations, we hypothesized that PTPN2 maintains normal mitochondrial morphology and function by suppressing TFRC. Consistent with this hypothesis, a significant increase in TFRC mRNA (Figure 3C) and protein expression (Figure 3D) levels was detected following PTPN2 knockout. Furthermore, IHC staining of subcutaneous tumors from ALK^+^ ALCL mouse xenograft models revealed strong positive TFRC expression in the PTPN2 knockout group (Figure 3E). In contrast, TFRC expression remained unchanged in the negative control FARAGE after PTPN2 knockout, highlighting the specificity of this regulatory mechanism in ALK^+^ ALCL (Figure S3A–C, Supporting Information).

To further elucidate the regulatory mechanism of PTPN2 on TFRC, we introduced MG132 and cycloheximide (CHX) to inhibit proteasomal degradation and protein synthesis, respectively. TFRC expression in PTPN2‐deficient cells remained unchanged upon MG132 treatment but significantly decreased with CHX, indicating PTPN2 modulates TFRC expression at the transcriptional level rather than through post‐translational regulation of protein stability (Figure 3F; Figure S3D, Supporting Information). Moreover, no PTPN2‐TFRC binding was detected by co‐immunoprecipitation (Co‐IP) experiments, suggesting PTPN2 regulates TFRC indirectly through transcriptional mechanisms rather than protein‐protein interactions (Figure 3G). Next, we utilized prediction databases (hTFtarget, KnockTF, and ENCODE) to identify potential transcription factors regulating TFRC, narrowing down the candidates to 15 transcription factors (HIF1A, CEBPB, GATA1, SIX5, SP1, STAT1, RFX5, TEAD4, NRF1, FOXA1, FOXM1, JUN, TFAP2C, USF2, CREB1) and hypoxia‐inducible factor 1 alpha (HIF1A) was identified as the potential transcription factor combined with RNA‐seq in Karpas299 (Figure 3H). To confirm the direct interaction between the above transcription factors and TFRC promoter, we performed chromatin immunoprecipitation followed by quantitative polymerase chain reaction (ChIP‐qPCR) assays in ALK^+^ ALCL cells. The results confirmed the direct binding of HIF1A, SP1, NRF1, and FOXM1 to the −2000 bp to −1 bp region of TFRC promoter (Figure 3I; Figure S3E, Supporting Information). Subsequent quantitative reverse transcription polymerase chain reaction (RT‐qPCR) assay revealed an increase in HIF1A mRNA levels while no change in SP1, NRF1, and FOXM1 in ALK^+^ ALCL cell lines following PTPN2 knockout (Figure S3F, Supporting Information). Moreover, a significant increase in HIF1A expression was observed after PTPN2 knockout (Figure 3J) and a reduction in TFRC expression upon HIF1A knockout (Figure 3K). We also performed experiments on FARAGE. While ChIP‐qPCR analysis showed the presence of PTPN2 and TFRC binding, PTPN2 was not involved in the regulation of TFRC in FARAGE (Figure S3G–I, Supporting Information).

Collectively, these findings indicated that PTPN2 repressed TFRC transcription by targeting the transcription factor HIF1A. This regulatory mechanism provided novel insights into the role of PTPN2 in mitochondrial function and its potential implications in tumorigenesis.

### 2.4 PTPN2 Drives Tumorigenesis via TFRC‐Mediated Mitophagy in ALK^+^ ALCL, Independent of Ferroptosis

**Figure 4:**
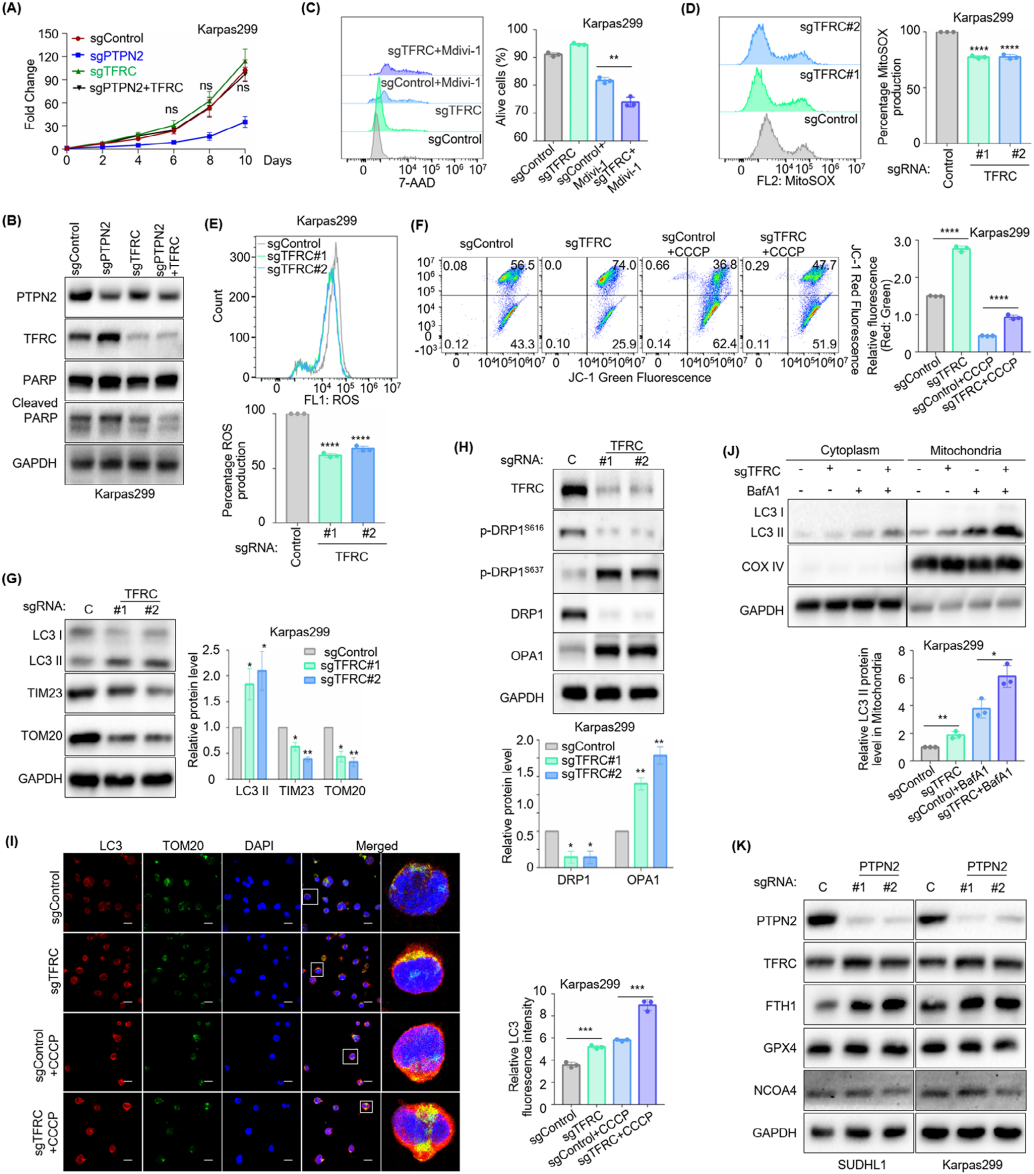
PTPN2 drives tumorigenesis via TFRC‐mediated mitophagy in ALK^+^ ALCL, independent of ferroptosis. A) Growth curve analysis showing the proliferation rate of Cas9+ Karpas299 following control, PTPN2 or TFRC targeting sgRNAs expression. *n* = 3. B) Immunoblot analysis showing the expression of PTPN2, TFRC, PARP, and cleaved PARP in Cas9+ Karpas299 following control, PTPN2 or TFRC targeting sgRNAs expression. C) Flow cytometry analysis showing cell death in Cas9+ Karpas299 that express control or TFRC sgRNAs with 10 µm mdivi‐1 for 48 h using 7‐AAD Kit. *n* = 3. D) MitoSOX analysis in Cas9+ Karpas299 that express control or TFRC sgRNAs. *n* = 3. E) ROS analysis in Cas9+ Karpas299 that express control or TFRC sgRNAs. *n* = 3. F) Mitochondrial membrane potential analysis in Cas9+ Karpas299 that express control or TFRC sgRNAs without or with 10 µm CCCP for 24 h by JC‐1. *n* = 3. G) Immunoblot analysis showing the expression of LC3 I/II, TIM23, and TOM20 in Cas9+ Karpas299 that express control (C) or TFRC sgRNAs. *n* = 3. H) Immunoblot analysis showing the expression of p‐DRP1^S616^, p‐DRP1^S637^, DRP1, and OPA1 in Cas9+ Karpas299 that express control (C) or TFRC sgRNAs. n = 3. I) Immunofluorescence analysis showing LC3 expression around mitochondria in Cas9+ Karpas299 that express control or TFRC sgRNAs without or with 10 µm CCCP for 24 h. Scale bar: 20 µm. *n* = 3. J) Immunoblot analysis showing LC3 I/II expression around mitochondria in Cas9+ Karpas299 that express control or TFRC sgRNAs without or with 20 nm Bafilomycin A1 (BafA1) for 24 h. *n* = 3. K) Immunoblot analysis showing the expression of TFRC, FTH1, GPX4, and NCOA4 in Cas9+ SUDHL1 and Karpas299 that express control (C) or PTPN2 sgRNAs. The data are shown as the mean ± SDs. ns, no significant; *, *p* < 0.05; **, *p* < 0.01; ***, *p* < 0.001; ****, *p* < 0.0001. Statistical analysis in panels A, and C‐J was performed by one‐way ANOVA with multiple comparisons.

To further explore whether TFRC plays an oncogenic role in ALK^+^ ALCL, control, PTPN2, or TFRC targeting sgRNAs were expressed in ALK^+^ ALCL cell lines. Functional assays revealed that TFRC knockout restored the decreased proliferation rate (**Figure** **4**A; Figure S4A, Supporting Information) and the increased apoptosis‐related protein (the cleaved forms of PARP) expression (Figure 4B; Figure S4B, Supporting Information) induced by PTPN2 knockout, indicating that PTPN2 promotes ALK^+^ ALCL progression by inhibiting TFRC.

Further investigation was conducted on the role of TFRC in mitophagy. We observed that 7‐AAD uptake was significantly increased in TFRC‐deficient ALK^+^ ALCL cells upon the treatment of mitochondrial division inhibitor mdivi‐1, suggesting that TFRC‐mediated mitophagy plays a protective role (Figure 4C; Figure S4C, Supporting Information). Mitochondrial changes were also assessed upon TFRC knockout. In contrast to PTPN2 depletion, TFRC knockout reduced mitoSOX levels (Figure 4D; Figure S4D, Supporting Information), decreased ROS production (Figure 4E; Figure S4E, Supporting Information), increased MMP (Figure 4F; Figure S4F, Supporting Information), and attenuated the accumulation of damaged mitochondria induced by CCCP treatment (Figure 4F; Figure S4F, Supporting Information) in ALK^+^ ALCL cells. Notably, the effects on mitophagy morphology and function induced by PTPN2 silencing could be restored by TFRC knockout (Figure S4G–L, Supporting Information). Furthermore, TFRC silencing upregulated LC3 II expression accompanied by reduced TIM23 and TOM20 expression (Figure 4G; Figure S5A, Supporting Information). The expression of p‐DRP1^S616^ and DRP1 were downregulated while p‐DRP1^S637^ and OPA1 expression were upregulated in TFRC‐deficient ALK^+^ ALCL cells (Figure 4H; Figure S5B, Supporting Information). Moreover, TFRC silencing enhanced the mitochondrial localization of LC3 upon CCCP treatment (Figure 4I; Figure S5C, Supporting Information). LC3 II accumulation induced by BafA1 was further exacerbated following TFRC knockout (Figure 4J; Figure S5D, Supporting Information). Collectively, these results suggested that TFRC plays a disruptive role in mitochondrial function and mitophagy and PTPN2 maintains mitochondrial function by inhibiting TFRC expression.

Given the established role of TFRC in ferroptosis,^[^ ^20^ ^]^ ferroptosis‐related markers were analyzed. However, no significant changes were observed in ferritin heavy chain 1 (FTH1), glutathione peroxidase 4 (GPX4), or nuclear receptor coactivator 4 (NCOA4) following PTPN2 knockout (Figure 4K). Overall, the absence of PTPN2 led to the inhibition of mitophagy and impaired clearance of damaged mitochondria by disinhibiting TFRC expression, ultimately resulting in cell apoptosis in ALK^+^ ALCL. This process functioned independently of ferroptosis.

### 2.5 PTPN2 Enhances PINK1‐PRKN‐Dependent Mitophagy by Suppressing TFRC Expression

**Figure 5:**
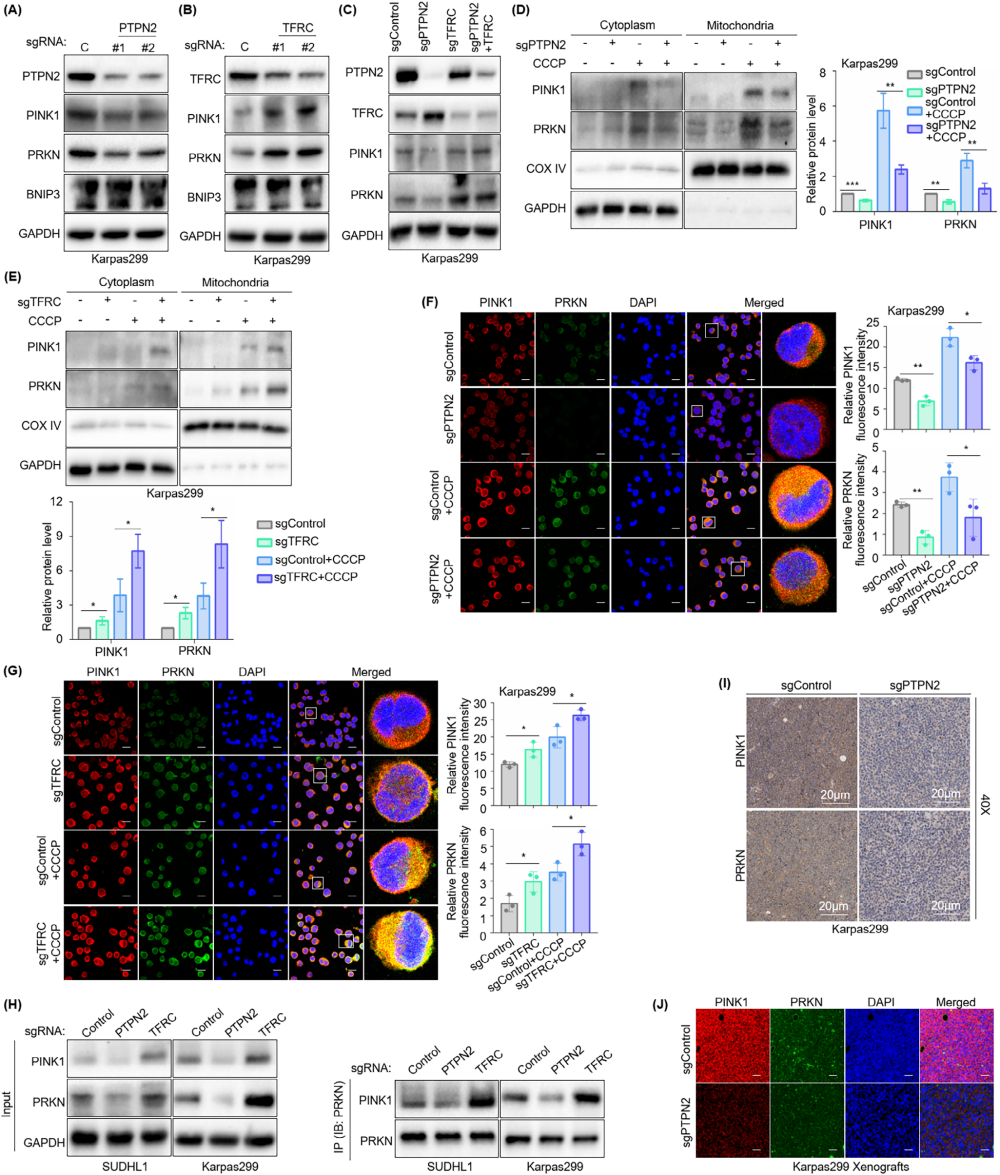
PTPN2 enhances PINK1‐PRKN‐dependent mitophagy by suppressing TFRC Expression. A) Immunoblot analysis showing the expression of PINK1, PRKN, and BNIP3 in Cas9+ Karpas299 that express control (C) or PTPN2 sgRNAs. B) Immunoblot analysis showing the expression of PINK1, PRKN, and BNIP3 in Cas9+ Karpas299 that express control or TFRC sgRNAs. C) Immunoblot analysis showing the expression of PTPN2, TFRC, PINK1, and PRKN in Cas9+ Karpas299 following control, PTPN2 or TFRC targeting sgRNAs expression. D) Immunoblot analysis showing PINK1 and PRKN expression in the mitochondria of Cas9+ Karpas299 that express control or PTPN2 sgRNAs without or with 10 µm CCCP for 24 h. *n* = 3. E) Immunoblot analysis showing PINK1 and PRKN expression in the mitochondria of Cas9+ Karpas299 that express control or TFRC sgRNAs without or with 10 µm CCCP for 24 h. *n* = 3. F) Immunofluorescence analysis showing PINK1 and PRKN expression around mitochondria in Cas9+ Karpas299 that express control or PTPN2 sgRNAs without or with 10 µm CCCP for 24 h. Scale bar: 20 µm. *n* = 3. G) Immunofluorescence analysis showing PINK1 and PRKN expression around mitochondria in Cas9+ Karpas299 that express control or TFRC sgRNAs without or with 10 µm CCCP for 24 h. Scale bar: 20 µm. *n* = 3. H) Co‐IP assay showing the bindings between PINK1 protein and PRKN protein in Cas9+ SUDHL1 and Karpas299 that express control or TFRC sgRNAs. I,J) Immunohistochemistry staining assay (I) and immunofluorescence analysis (J) showing PINK1 and PRKN expression in xenograft mouse models constructed using Cas9+ Karpas299 following control or PTPN2 targeting sgRNAs expression. Scale bar: 20 µm. *n* = 5. The data are shown as the mean ± SDs. *, *p* < 0.05; **, *p* < 0.01; ***, *p* < 0.001; ****, *p* < 0.0001. Statistical analysis in panels D‐G was performed by one‐way ANOVA with multiple comparisons.

Mitophagy regulatory systems mainly include the PINK1 (PTEN induced kinase 1) ‐PRKN (parkin RBR E3 ubiquitin protein ligase) ‐mediated ubiquitin‐dependent pathway and the BCL2 interacting protein 3 (BNIP3) ‐mediated ubiquitin‐independent pathway.^[^ ^21^ ^]^ Down‐regulated expression of PINK1 and PRKN, but not BNIP3, was observed following PTPN2 knockout (**Figure** **5**A; Figure S5E, Supporting Information). Conversely, TFRC knockout led to upregulated expression of PINK1 and PRKN, with no significant change in BNIP3 expression (Figure 5B; Figure S5F, Supporting Information). Furthermore, the inhibitory effect of PTPN2 deficiency on PINK1‐PRKN signaling was eliminated upon TFRC knockout (Figure 5C; Figure S5G, Supporting Information). Therefore, it was presumed that inhibition of PTPN2 disrupted PINK1‐PRKN‐mediated mitophagy by disinhibition of TFRC.

To further verify, we introduced CCCP to induce PINK1‐PRKN‐dependent mitophagy.^[^ ^22^ ^]^ PTPN2 knockout significantly mitigated the accumulation of PINK1 and PRKN induced by CCCP in mitochondria (Figure 5D), while TFRC knockout aggravated the accumulation (Figure 5E). The colocalization of PINK1 and PRKN within mitochondria was diminished upon PTPN2 knockout (Figure 5F) but enhanced following TFRC knockout (Figure 5G). Additionally, Co‐IP assay revealed reduced binding between PINK1 and PRKN after PTPN2 knockout and increased binding upon TFRC knockout (Figure 5H). The aforementioned findings were validated in vivo. IHC staining assay (Figure 5I) showed weak positivity of PINK1 and PRKN expression, and IF analysis (Figure 5J) confirmed decreased colocalization of PINK1 and PRKN in the PTPN2 knockout group of the subcutaneous tumors from ALK^+^ ALCL mouse xenograft models. Collectively, these findings demonstrated that the absence of PTPN2 suppressed TFRC‐mediated PINK1‐PRKN‐dependent mitophagy in ALK^+^ ALCL.

### 2.6 PTPN2/N1 Inhibitor AC484 Disrupts Mitochondrial Function to Exerts Anti‐Tumor Effects

**Figure 6:**
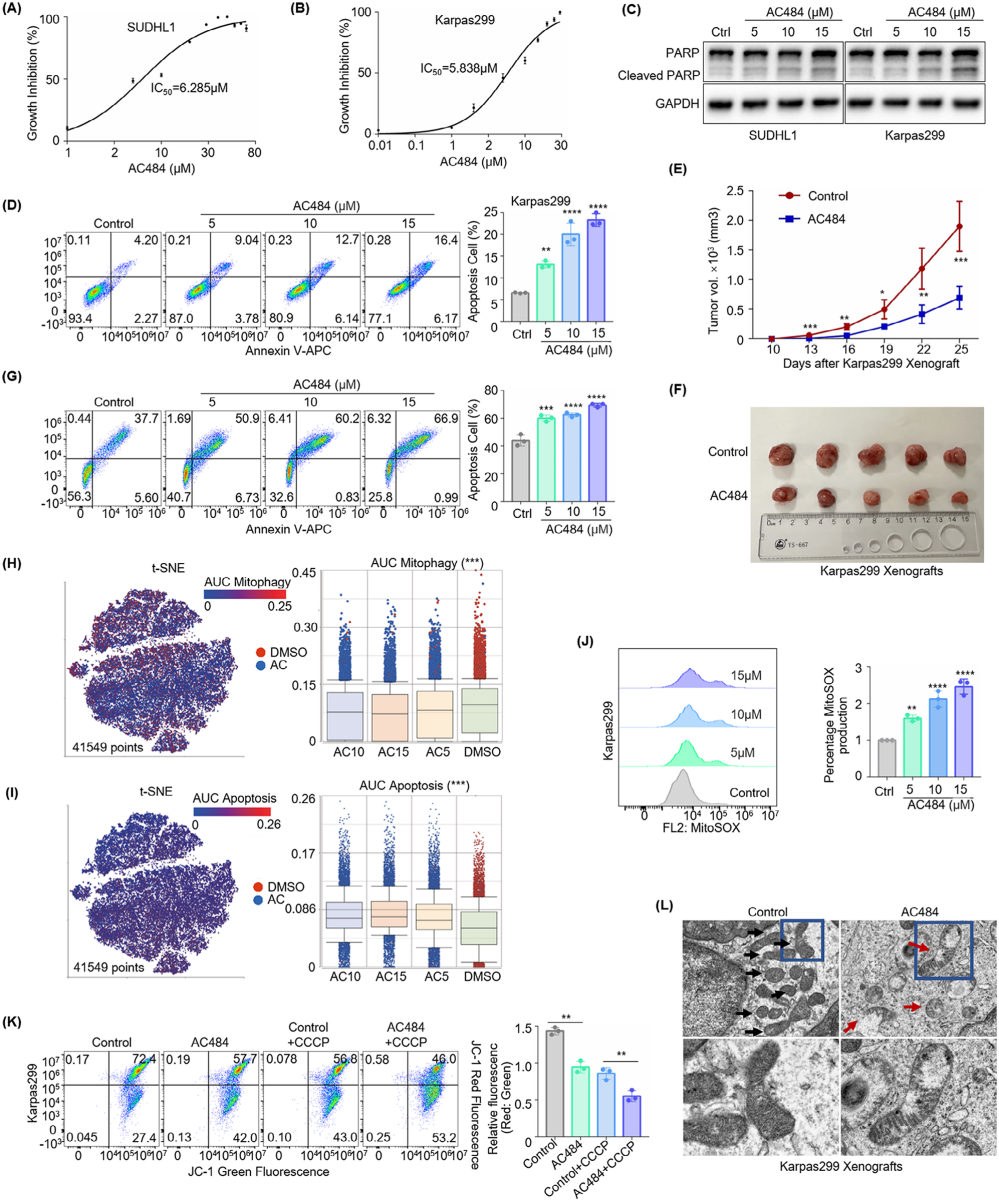
PTPN2/N1 inhibitor AC484 disrupts mitochondrial function to exert anti‐tumor effects. A, B) CCK8 analysis showing the 72 h‐IC50 of AC484 in SUDHL1 (A) and Karpas299 (B). *n* = 3. C) Immunoblot analysis showing PARP and cleaved PARP expression in SUDHL1 (left) and Karpas299 (right) treated with 5,10,15 µm AC484 for 48 h. D) Flow cytometry analysis showing cell apoptosis in Karpas299 treated with 5,10,15 µm AC484 for 48 h using 7‐AAD Kit. *n* = 3. E,F) Growth curve (E) and volume analysis (F) showing tumor proliferation and size in Karpas299 xenograft mouse models following AC484 administration at the daily dose of 3 mg kg^−1^ for 15 days. *n* = 5 per group. G) Flow cytometry analysis showing cell apoptosis in the primary tumor cells, which were isolated from the hydrothorax of one ALK^+^ ALCL patient using annexin V‐APC/7‐AAD Kit. *n* = 3. H, I) AUCell analysis showing the ‘Mitophagy’ activity (H) and the ‘Apoptosis’ activity (I) in Karpas299 treated with 5,10,15 µm AC484 for 72 h by single‐cell RNA sequencing. J) MitoSOX analysis in Karpas299 treated with 5,10,15 µm AC484 for 48 h. *n* = 3. K) Mitochondrial membrane potential analysis in Karpas299 treated with 10 µm AC484 or/and 10 µm CCCP for 24 h by JC‐1. *n* = 3. L) Transmission electron microscopy analysis showing mitochondrial morphology in Karpas299 xenograft mouse models following AC484 administration at the daily dose of 3 mg kg^−1^ for 15 days. x10k. *n* = 5. The data are shown as the mean ± SDs. *, *p* < 0.05; **, *p* < 0.01; ***, *p* < 0.001; ****, *p* < 0.0001. Statistical analysis in panels D, E, G, and H‐K was performed by one‐way ANOVA with multiple comparisons.

We have demonstrated that PTPN2 promotes mitochondrial renewal through PINK1‐PRKN‐dependent mitophagy by suppressing TFRC expression, thereby exerting tumor‐promoting effects in ALK^+^ ALCL. Given the promising therapeutic potential of the oral PTPN2/N1 inhibitor AC484, we further investigated its efficacy against ALK^+^ ALCL. First, we determined the 50% inhibitory concentration (IC50) of AC484 for 72 h, which was estimated to be 6.285 µm in SUDHL1 cells (**Figure** **6**A) and 5.838 µm in Karpas299 cells (Figure 6B). With increasing concentrations of AC484, the expression of the cleaved forms of PARP rose accordingly (Figure 6C) and apoptosis gradually increased (Figure 6D; Figure S6A, Supporting Information) in ALK^+^ ALCL, but not in the negative control FARAGE (Figure S6B,C, Supporting Information). Moreover, AC484 treatment was not significantly functional in the PTPN2‐deficient ALK^+^ ALCL cells (Figure S6D–F, Supporting Information). In vivo experiments were also conducted. We tested the anti‐tumor effects of AC484 in Karpas299 mouse xenograft models (*n* = 5 per group). Ten days after subcutaneous cell injection, AC484 was administered at the daily dose of 3 mg kg^−1^ for 15 days. The mice in the AC484‐treated group showed significant tumor growth rate (Figure 6E) and tumor size (Figure 6F) regression. Additionally, we evaluated the effect of AC484 on primary tumor cells isolated from the pleural effusion of an ALK^+^ ALCL patient and observed that AC484 markedly increased apoptotic cells (Figure 6G). The flow cytometric results of the pleural effusion were presented in Figure S7 (Supporting Information). Collectively, these results demonstrated that AC484 induced apoptosis and inhibited tumor growth in ALK^+^ ALCL, highlighting its therapeutic potential for ALK^+^ ALCL patients.

To further explore the therapeutic potential of AC484 in ALK^+^ ALCL, we found the negative effects of AC484 in the mitochondria. Single‐cell RNA sequencing was performed on Karpas299 cells treated with 5,10,15 µm AC484 for 72 h. Following quality control and unsupervised clustering, t‐stochastic neighbor embedding (t‐SNE) analysis visualized 41,549 cells labeled by different concentrations of AC484 (*n* = 4) (Figure S8A, Supporting Information). AUCell analysis revealed that the ′Mitophagy′ activity scores were significantly lower in AC484‐treated groups, indicating impaired mitophagy activation (Figure 6H). Additionally, we assessed the activities of common cell death pathways and observed a marked increase in ′Apoptosis′ scores in AC484‐treated groups (Figure 6I, Figure S8B‐I, Supporting Information). Flow cytometric analysis showed that AC484 significantly increased mitoSOX generation (Figure 6J, Figure S9A, Supporting Information) and reduced MMP levels (Figure S9B,C, Supporting Information). Furthermore, AC484 exacerbated mitochondrial damage induced by CCCP (Figure 6K; Figure S9D, Supporting Information). Notably, the effects of AC484 on mitophagy morphology and function did not work following PTPN2 knockout (Figure S9E–I, Supporting Information). In the subcutaneous tumors of a Karpas299 mouse xenograft model, TEM analysis also observed damaged swelling mitochondria aggregated in tumor cells treated with AC484 (Figure 6L). Taken together, these findings highlighted the negative effects of AC484 on mitochondrial function and its potential as a therapeutic agent in ALK^+^ ALCL.

### 2.7 AC484 Disturbs Mitochondrial Renewal and Blocks TFRC‐Mediated PINK1‐PRKN‐Dependent Mitophagy

**Figure 7:**
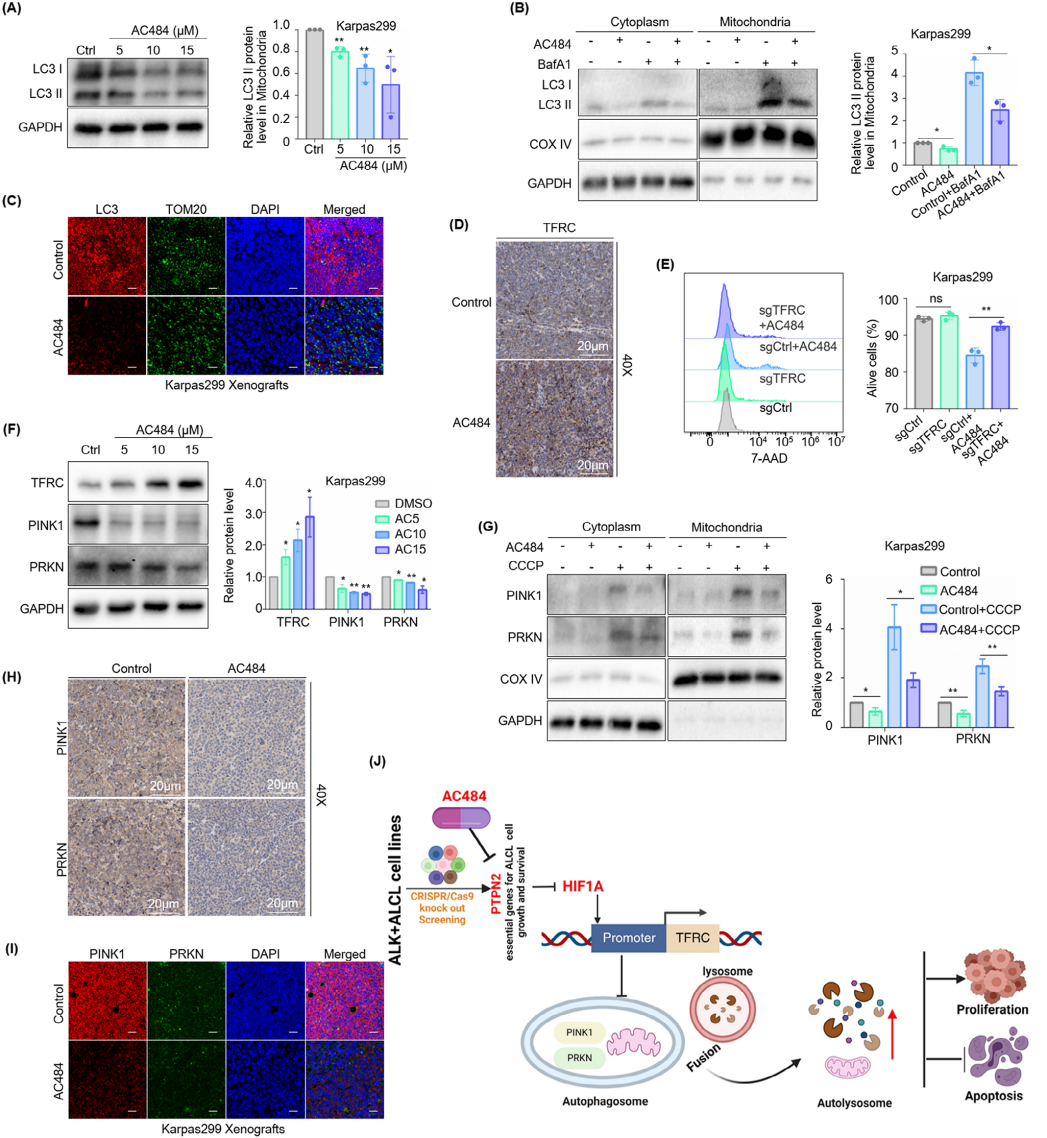
AC484 disturbs mitochondrial renewal and blocks TFRC‐mediated PINK1‐PRKN‐dependent mitophagy. A) Immunoblot analysis showing LC3 I/II expression in Karpas299 treated with 5,10,15 µm AC484 for 48 h. *n* = 3. B) Immunoblot analysis showing LC3 I/II expression in the mitochondria of Karpas299 treated with 10 µm AC484 and/or 20 nm Bafilomycin A1 (BafA1) for 24 h. *n* = 3. C) Immunofluorescence analysis showing LC3 expression around mitochondria in Karpas299 xenograft mouse models following AC484 administration at the daily dose of 3 mg kg^−1^ for 15 days. Scale bar: 20 µm. *n* = 5. D) Immunohistochemistry staining assay showing TFRC expression in Karpas299 xenograft mouse models following AC484 administration at the daily dose of 3 mg kg^−1^ for 15 days. Scale bar: 20 µm. *n* = 5. E) Flow cytometry analysis showing cell death in Karpas299 that express control or TFRC sgRNAs with or without 10 µm AC484 or/and 10 µm mdivi‐1 for 48 h using 7‐AAD Kit. *n* = 3. F) Immunoblot analysis showing the expression of TFRC, PINK1, and PRKN in Karpas299 treated with 5,10,15 µm AC484 for 48 h. *n* = 3. G) Immunoblot analysis showing PINK1 and PRKN expression in the mitochondria of Karpas299 treated with 10 µm AC484 or/and 10 µm CCCP for 24 h. *n* = 3. H, I) Immunohistochemistry staining assay (H) and immunofluorescence analysis (I) showing PINK1 and PRKN expression in Karpas299 xenograft mouse models following AC484 administration at the daily dose of 3 mg kg^−1^ for 15 days. Scale bar: 20 µm. *n* = 5. J) Schematic model of PTPN2 effects on ALK^+^ ALCL. PTPN2 expresses highly and promotes TFRC‐mediated PINK1‐PRKN‐dependent mitophagy by regulating transcription factor HIF1A negatively to play cancer‐promoting effects in ALK^+^ ALCL. The first‐in‐class PTPN2/N1 inhibitor AC484 disturbs mitochondrial renewal and blocks TFRC‐mediated PINK1‐PRKN‐dependent mitophagy, leading to damaged mitochondria accumulation and cell apoptosis to exert anti‐tumor activities in ALK^+^ ALCL. The data are shown as the mean ± SDs. ns, no significant; *, *p* < 0.05; **, *p* < 0.01; ***, *p* < 0.001; ****, *p* < 0.0001. Statistical analysis in panels A, B, and E‐G was performed by one‐way ANOVA with multiple comparisons.

We next investigated the effects of AC484 on mitophagy. Treatment with AC484 significantly reduced LC3 II expression (**Figure** **7**A; Figure S9J, Supporting Information) and attenuated the aggregation of LC3 induced by BafA1 (Figure 7B), but not in the negative control FARAGE (Figure S9K, Supporting Information). Moreover, LC3 expression around mitochondria was markedly decreased in the subcutaneous tumors of the AC484‐treated group (Figure 7C).

Further experiments demonstrated that AC484 exerted anti‐tumor activities by TFRC‐mediated PINK1‐PRKN‐dependent mitophagy. IHC analysis revealed strong TFRC staining in subcutaneous tumors from the AC484‐treated group (Figure 7D). Additionally, TFRC deficiency diminished cell death induced by AC484 (Figure 7E; Figure S9L, Supporting Information). Immunoblot blot analysis showed that AC484 treatment inhibited PINK1 and PRKN expression with an increase in TFRC expression (Figure 7F; Figure S9M, Supporting Information), and the accumulation of PINK1 and PRKN induced by CCCP in mitochondria (Figure 7G). In vivo, AC484 treatment reduced PINK1 and PRKN expression (Figure 7H) and the colocalization of PINK1 and PRKN in mitochondria (Figure 7I) in the subcutaneous tumors from the AC484‐treated group. Collectively, these results demonstrated that AC484 exerted anti‐tumor effects by modulating TFRC expression, which in turn disrupted PINK1‐PRKN‐dependent mitophagy.

## 3 Discussion

PTPN2 participates in multiple pathological processes, including inflammatory reaction, tumorigenesis, and immune regulation.^[^ ^5^ ^]^ Multiple studies demonstrated PTPN2 as a tumor suppressor in human T‐cell acute lymphoblastic leukemia (T‐ALL).^[^ ^23^ ^]^ PTPN2 inactivation was reported in 2 out of 39 (5%) cases of peripheral T‐cell lymphoma not otherwise specified (PTCL, NOS).^[^ ^24^ ^]^ In contrast, Prutsch N et al. have identified a small group of genes including PTPN2, BATF3, IRF4, IKZF1, STAT3, and SBNO2 as a selective dependency in ALK^+^ ALCL cell lines compared to other cancer cells by analyzing DepMap data, which was consistent with our research results.^[^ ^25^ ^]^ A genome‐wide screening study by Chiarle's team has also identified PTPN2 as a vulnerability in ALK inhibitor crizotinib resistance by attenuating ALK phosphorylation.^[^ ^26^ ^]^ In our study, we demonstrated the tumor‐promoting role of PTPN2 through engineering the loss‐of‐function models by CRISPR/Cas9 editing in vivo and in vitro, highlighting the specific oncogenic role of PTPN2 in ALK^+^ ALCL. These findings aligned with Ng SY. et al. ′s^[^ ^10^ ^]^ identification of PTPN2 as a specific vulnerability in ALK^+^ ALCL cell line KIJK. These different research results may reflect context‐dependent roles of PTPN2. We emphasized the role of PTPN2 in the regulation of mitochondrial turnover, while Chiarle's work underscored its modulation of oncogenic ALK‐STAT3 signaling. These findings emphasize the multifaceted roles of PTPN2 in tumor development and warrant further investigation to fully elucidate its mechanisms and therapeutic potential.

Intracellular iron homeostasis plays important oncogenic roles in hematologic malignancies.^[^ ^27^ ^]^ TFRC on the cell surface transfers extracellular iron into the cell cytosol to maintain intracellular iron homeostasis following combing with transferrin (TF).^[^ ^12^, ^28^ ^]^ While TFRC's role in ferroptosis is well‐established,^[^ ^20^, ^29^ ^]^ a study from the German Cancer Research Center revealed a distinct effect of TFRC in regulating mitochondrial morphology and function.^[^ ^13^ ^]^ Inspired by this finding, we proposed that PTPN2 deficiency disrupts PINK1‐PRKN‐mediated mitophagy and impairs mitochondrial clearance by disinhibiting TFRC expression. NCOA4 targets cytosolic iron storage protein ferritin to lysosomes for ferritin degradation and iron release via autophagy, referred to as ferritinophagy.^[^ ^30^ ^]^ Our findings revealed no changes in ferroptosis‐ and ferritinophagy‐related protein FTH1, GPX4, and NCOA4 following PTPN2 knockout, indicating TFRC‐mediated mitophagy in ALK^+^ ALCL was ferroptosis‐independent. While our findings enhance the understanding of TFRC's role in mitochondrial function, the precise molecular mechanisms remain elusive and warrant further investigation.

Just half of patients with relapsed/refractory ALK^+^ ALCL achieve CR after second‐line treatment.^[^ ^4a^ ^]^ Manguso RT et al. demonstrated that PTPN2 deletion markedly enhanced immunotherapy response through pooled loss‐of‐function genetic screens in vivo.^[^ ^5c^ ^]^ Similarly, LaFleur MW et al. reported that PTPN2 loss in CD8^+^ T cells promoted anti‐tumor immunity.^[^ ^31^ ^]^ Consistent with these findings, our study has identified PTPN2 as a cancer‐promoting factor, providing a novel therapeutic target for ALK^+^ ALCL. Currently, a phase I clinical trial of the first‐in‐class PTPN2/N1 inhibitor AC484 is in progress with advanced solid tumors (NCT04777994).^[^ ^11^ ^]^ In this study, AC484 demonstrated a great anti‐tumor effect against ALK^+^ ALCL. Furthermore, the validation experiments in vivo and in vitro revealed the underlying mechanism of AC484 in the disruption of mitochondrial function and TFRC‐mediated PINK1‐PRKN‐dependent mitophagy, providing the basis for future clinical trials of AC484 in ALK^+^ ALCL. Due to the restricted number of ALK^+^ ALCL models in the present study, there are still some limitations. To address this, we are actively pursuing a larger number of ALK^+^ ALCL models including KIJK, SUPM2, and DEL, and ALK‐negative ALCL models for further investigation. In‐depth research of PTPN2 and AC484 in ALCL are still being conducted in our laboratory.

Mitophagy regulatory systems are generally categorized into PINK1‐PRKN‐mediated ubiquitin‐dependent pathway and BNIP3‐mediated ubiquitin‐independent pathway.^[^ ^21^ ^]^ Our data revealed that PTPN2 deficiency disrupted PINK1‐PRKN‐mediated mitophagy by disinhibiting TFRC. The inhibition of mitophagy resulted in increased cellular apoptosis, implying a protective role of PINK1‐PRKN‐mediated mitophagy in ALK^+^ ALCL. Multiple studies have documented tumor suppression mechanisms of Parkin activity, or mitophagy.^[^ ^32^ ^]^ Therefore, targeting the disruption of PINK1‐PRKN‐mediated mitophagy may be a promising therapeutic strategy for ALK^+^ ALCL. Notably, we identified that HIF1A promotes the transcription of TFRC by binding to the promoter of TFRC. Notably, Martinengo C. et al.^[^ ^33^ ^]^ demonstrated that hypoxia signaling pathways were significantly activated in ALK^+^ ALCL, where ALK regulated this process through STAT3 activation. Their work further revealed that HIF2A, but not HIF1A, was essential for ALK^+^ ALCL tumor growth. These results reveal a striking divergence in HIF pathway functionality within ALK^+^ ALCL, highlighting the critical need for deeper mechanistic exploration.

In summary, we demonstrate that PTPN2 exerts cancer‐promoting effects by TFRC‐mediated mitophagy, a process that is independent of ferroptosis. Mechanistically, PTPN2 negatively regulates TFRC expression via the transcription factor HIF1A. PTPN2 deficiency results in PINK1‐PRKN‐mediated mitophagy inhibition and damaged mitochondria clearance through disinhibiting TFRC expression. Intriguingly, the first‐in‐class PTPN2/N1 inhibitor AC484 shows a great anti‐tumor effect against ALK^+^ ALCL, providing a rationale for conducting clinical trials of AC484 on ALK^+^ ALCL patients (Figure 7J).

## 4 Experimental section

### Patients and Cell Lines

ALK^+^ ALCL patient samples were collected from the First Affiliated Hospital with Nanjing Medical University with written informed consent approved by the Ethics Committee (approval number: 2023‐SR‐190). Peripheral blood mononuclear cells (PBMCs) were isolated from the peripheral blood of healthy donors. HEK293T, SUDHL1, RAJI, Namalwa, SUDHL8, and FARAGE were purchased from the American Type Culture Collection (ATCC) while Kapras299 was from Cobioer (Nanjing, China), Granta519 was from the German Collection of Microorganisms and Cell Cultures GmbH (DSMZ). Cells were cultured in RPMI‐1640 or IMDM medium (Gibco) containing 10% fetal bovine serum (VivaCell) and 1% penicillin‐streptomycin (Beyotime) with a humidified atmosphere containing 5% CO2 at 37 °C. Short tandem repeat (STR) profiling was examined in all cell lines. Growth curve analysis was performed with a cytometer (Countstar).

### DepMap Analysis

Gene effects of ALK^+^ ALCL cell lines, PTPN2 CRISPR (DepMap Public 24Q4 + Score Chronos), and expression data (Public 24Q4) were downloaded from DepMap portal.^[^ ^34^ ^]^ PTPN2 expression data were used to generate violin plots for different types of tumor cell lines, and PTPN2 CRISPR data were used to construct scatter plots to compare the gene effects of PTPN2 between ALK^+^ ALCL and other cancer cell lines using the limma analysis method.

### CRISPR/Cas9 Gene Editing

Gene knockout was achieved by CRISPR/Cas9 system.^[^ ^35^ ^]^ Two sgRNAs were designed using the Benchling CRISPR design tool (Table S3, Supporting Information) and inserted into lentiGuide‐Puro (Addgene) or pLenti SpBsmBI sgRNA Hygro (Addgene). Lentiviruses were generated by HEK293T transfection.

### Xenograft Study

All animal studies were approved by the Institutional Animal Care and Use Committee of Nanjing Medical University and in accordance with the NIH Guide for Care and Use of Laboratory Animals (approval number: IACUC‐2401008). NOD/SCID male mice were purchased from GemPharmatech (China). Approximately 1 × 10^7^ wild‐type (WT) or PTPN2 knockout ALK^+^ ALCL cells were injected subcutaneously into the right anterior axilla. AC484 (MCE, HY‐145923) was dissolved and formulated in DMSO, PEG300, Tween‐80, and saline at a ratio of 10:40:5:45, respectively. Ten days after injection, AC484 was administered once a day at 3 mg kg^−1^ for 15 days, by gavage. Tumor volumes were measured on alternate days and calculated as (tumor length × tumor width^2^)/2.

### Transmission Electron Microscopy (TEM)

The fresh subcutaneous tumors of SUDHL1 and Karpas299 mouse xenograft models were cut and fixed with electron microscopy fixative (Servicebio, G1102) as described previously.^[^ ^15d^ ^]^ Mitochondrial morphology images were captured by HITACHI (HT7800/HT7700). Specific technical support was provided by Servicebio (China).

### RNA Sequencing (RNA‐seq) and Single‐Cell RNA Sequencing

RNA‐seq of PTPN2 knockout was performed in Karpas299 by Novogene (China) with bcl2fastq v2.19.0.316 (Illumina).^[^ ^36^ ^]^ Single‐cell RNA sequencing was performed in Karpas299 treated with 5, 10, 15 µM AC484 for 72 h by Singleron Biotechnologies (China) with GEXSCOPE Single‐Cell RNA Library Kit as previously reported.^[^ ^37^ ^]^

### Statistical Analysis

Statistical analysis was performed using GraphPad Prism 9.0 (La Jolla, CA, USA) and R software (Version 4.1.3). All experiments were performed at least three times. Data were presented as mean ± standard deviation (SD). *p* < 0.05 was considered significant. PTPN2 gene effect scores and expression data in all different types of tumor cell lines were obtained from DepMap (https://depmap.org/portal/). The limma analysis method was used to identify PTPN2 dependencies specific to ALK^+^ ALCL compared to other types of cancer cell lines using PTPN2 gene effect scores data from DepMap. Unpaired student's t‐test was used for comparisons between two groups, and ANOVA was used for multiple comparisons between groups. DEGs, KEGG analysis, and heatmap Hiplot (https://hiplot‐academic.com/) was used to visualize the results, including DEGs, KEGG analysis, and heatmap.

## Conflict of Interest

The authors declare no conflict of interest.

## Author Contributions

W.‐T.W. and Z.‐W.D. contributed equally to this work. W.‐T.W. and Z.‐W.D. performed experiments and wrote the manuscript. T.‐Y.X. provided sequencing data. W.H., K.‐X.D., C.‐Y.S., Y.‐F.W., L.W., and J.‐Y.L. collected and analyzed data. W.X., J.‐H.L., and R.G. conceived experiments, drafted, and revised the paper.

## Supporting information



Supporting Information

Supporting Data

## Data Availability

The data that support the findings of this study are available from the corresponding author upon reasonable request.
