# Supplementary material for: PTPN2 Inhibition Disrupts Mitochondrial Renewal and Blocks TFRC‐Mediated Mitophagy to Exert Anti‐Tumor Activities in ALK‐Positive Anaplastic Large Cell Lymphoma
Source: Adv Sci (Weinh). 2025 Jul 30;12(31):e14282. doi: 10.1002/advs.202414282 (PMC12376625; doi:10.1002/advs.202414282)
Supplement: Supplementary file 1 — Supporting Information [file ADVS-12-e14282-s002.docx]

**PTPN2 Inhibition Disrupts Mitochondrial Renewal and Blocks TFRC-mediated Mitophagy to Exert Anti-tumor Activities in ALK-positive Anaplastic Large Cell Lymphoma.**

**Supplemental Methods**

**Peripheral blood mononuclear cell isolation**

Peripheral blood mononuclear cells (PBMCs) were isolated from peripheral blood samples collected in EDTA-coated tubes using lymphocyte separation medium (Fcmacs Biotech, FMS-900013) according to the instructions. Freshly isolated PBMCs (≥90% viability) were labeled with anti-CD3 MicroBeads (Miltenyi Biotec, 130-097-043) in PBS containing 0.5% bovine serum albumin and 2 mM EDTA for 15 min at 4°C. Magnetically labeled cells were then separated using LS columns (Miltenyi Biotec) according to the manufacturer's protocol. The purity of isolated CD3-positive T lymphocytes was confirmed to be ≥95% by flow cytometry using CD3-FITC staining, with ≥98% viability.

**Immunohistochemistry (IHC)**

IHC was performed on formalin-fixed, paraffin-embedded (FFPE) sections of human lymph node biopsy samples from newly diagnosed ALK-positive anaplastic large cell lymphoma (ALK^+^ ALCL) before treatment and xenograft tumor tissues obtained from mice as previously described.^[1]^ The primary antibodies were against ALK-1 (Abcam, ab51870), PTPN2 (Abcam, ab314496), TFRC (Abcam, ab214039), PINK1 (Proteintech, 23274-1-AP), and PRKN (Proteintech, 14060-1-AP). For ALK/PTPN2 double staining, the tissue sections were first baked at 60-65°C for 60 min, followed by dewaxing in xylene (I, II, III) and hydration in an alcohol gradient (100%, 95%, 75%). Endogenous peroxidase was blocked (5 min, RT), and antigen retrieval was performed in boiling buffer (15-20 min). After marking water-repellent areas, primary antibody incubation (30 min, RT) and AP polymer/HRP polymer reactions (20 min/15 min, RT) were conducted, with AP-Red (20-30 min) and DAB (3-5 min) chromogen applications. Counterstaining with hematoxylin (1-2 min), bluing, dehydration (75%, 95%, 100% alcohol), and mounting completed the protocol. Images were acquired by ScanScope CS2 (Leica Biosystems).

**Quantitative reverse transcription polymerase chain reaction (RT-qPCR)**

Total RNA was extracted using an RNA Isolation Kit (Beyotime, R0077). Reverse Transcription kits (TaKaRa, RR036A) were used for cDNA synthesis, and SYBR Premix Ex Taq kits (TaKaRa, RR420A) were used for cDNA amplification. RT-qPCR primer sequences were listed in Table S4. ACTIN served as the reference gene and the results were analyzed by the comparative Ct (delta-delta-Ct, ΔΔCT) method.

**Flow cytometric analysis**

An annexin V-APC/7-AAD apoptosis detection Kit (KeyGEN BioTECH, Cat# KGA1023) was used for apoptosis and cell death assay. MitoSOX Red Mitochondrial Superoxide Indicator (Yeasen, 40778ES50) was used for mitochondrial superoxide (mitoSOX) assay. Reactive Oxygen Species (ROS) Assay Kit (Beyotime, S0033) was used for ROS assay. An enhanced mitochondrial membrane potential assay kit with JC-1 (Beyotime, C2003) was used to detect mitochondrial membrane potential (MMP) levels. All assays were processed according to the manufacturer’s instructions.

**Immunoblot and immunofluorescence (IF) analysis**

Immunoblot and IF were performed as previously described.^[2]^ The primary antibodies were against PTPN2 (CST, 58935), PARP (CST, 9542), CASPASE9 (CST, 9502), Cyclin D1 (Proteintech, 60186-1-Ig), LC3 (Proteintech, 14600-1-AP), TIM23 (CST, 34822), TOM20 (Proteintech, 66777-1-Ig), COX IV (CST, 4850), TFRC (Abcam, ab214039), HIF1A (CST, 36169), FTH1 (Santa Cruz, sc-376594), GPX4 (Abcam, ab125066), NCOA4 (CST, 66849), PINK1 (CST, 6946), PRKN (CST, 4211), BNIP3 (CST, 3769), DRP1 (CST, 8570), p‑DRP1^S616^ (CST, 3455), p‑DRP1^S637^ (CST, 4867), OPA1 (CST, 67589), GAPDH (Proteintech, 60004-1-Ig), and β-tubulin (Proteintech, 66240-1-Ig).

**Mitochondrial protein extraction**

Mitochondria were separated from whole cells using a Cell Mitochondria Isolation Kit (Beyotime, C3601) based on the protocol. The harvested mitochondria were lysed with RIPA lysis buffer. Then the mitochondrial protein was obtained following centrifugation at 12000 rpm for 10 min. Protein expression was detected by Immunoblot.

**Chromatin immunoprecipitation quantitative polymerase chain reaction (ChIP-qPCR) assay**

ChIP assay was performed using ChIP Kit (Absin, abs50034) based on the manufacturer’s instructions. The primary antibody was against HIF1A (Proteintech, 20960-1-AP), CEBPB (Proteintech, 23431-1-AP), GATA1 (Proteintech, 10917-2-AP), SIX5 (Proteintech, 22938-1-AP), SP1 (Proteintech, 21962-1-AP), STAT1 (Proteintech, 10144-2-AP), TEAD4 (Proteintech, 12418-1-AP), NRF1 (Proteintech, 12936-1-AP), FOXA1 (Proteintech, 20411-1-AP), FOXM1 (Proteintech, 13147-1-AP), JUN (Proteintech, 28891-1-AP), TFAP2C (Proteintech, 14572-1-AP), and CREB1 (Proteintech, 12208-1-AP). The forward primer of TFRC was AAACATTACGCAAAGCACTCC, and the reverse primer was TGACCTTGACCAACCTCCAG. Specific technical support was provided by Servicebio (China).

**Coimmunoprecipitation (Co-IP) assay**

An IP/Co-IP Kit (Absin, abs955) was used for Co-IP assay according to the instructions. Cell lysates were prepared and incubated with PRKN (Proteintech, 14060-1-AP), PTPN2 (Abcam, ab227916), or normal rabbit IgG (CST, 3900). The results were analyzed by Immunoblot.

**Chemical reagents**

CCCP (HY-100941), MG132 (HY-13259), cycloheximide (HY-12320), bafilomycin A1 (HY-100558), and mdivi-1 (HY-15886) were purchased from MCE.

**Reference**

[1] T. X. Lu, J. H. Liang, Y. Miao, L. Fan, L. Wang, X. Y. Qu, L. Cao, Q. X. Gong, Z. Wang, Z. H. Zhang, W. Xu, J. Y. Li, *Sci Rep* **2015**, *5*, 12168.

[2] W. T. Wang, T. Y. Xing, K. X. Du, W. Hua, J. R. Guo, Z. W. Duan, Y. F. Wu, J. Z. Wu, Y. Li, H. Yin, H. R. Shen, L. Wang, J. Y. Li, J. H. Liang, W. Xu, *Cancer Lett* **2024**, *583*, 216616.

**Supplemental Figures and Tables**

**
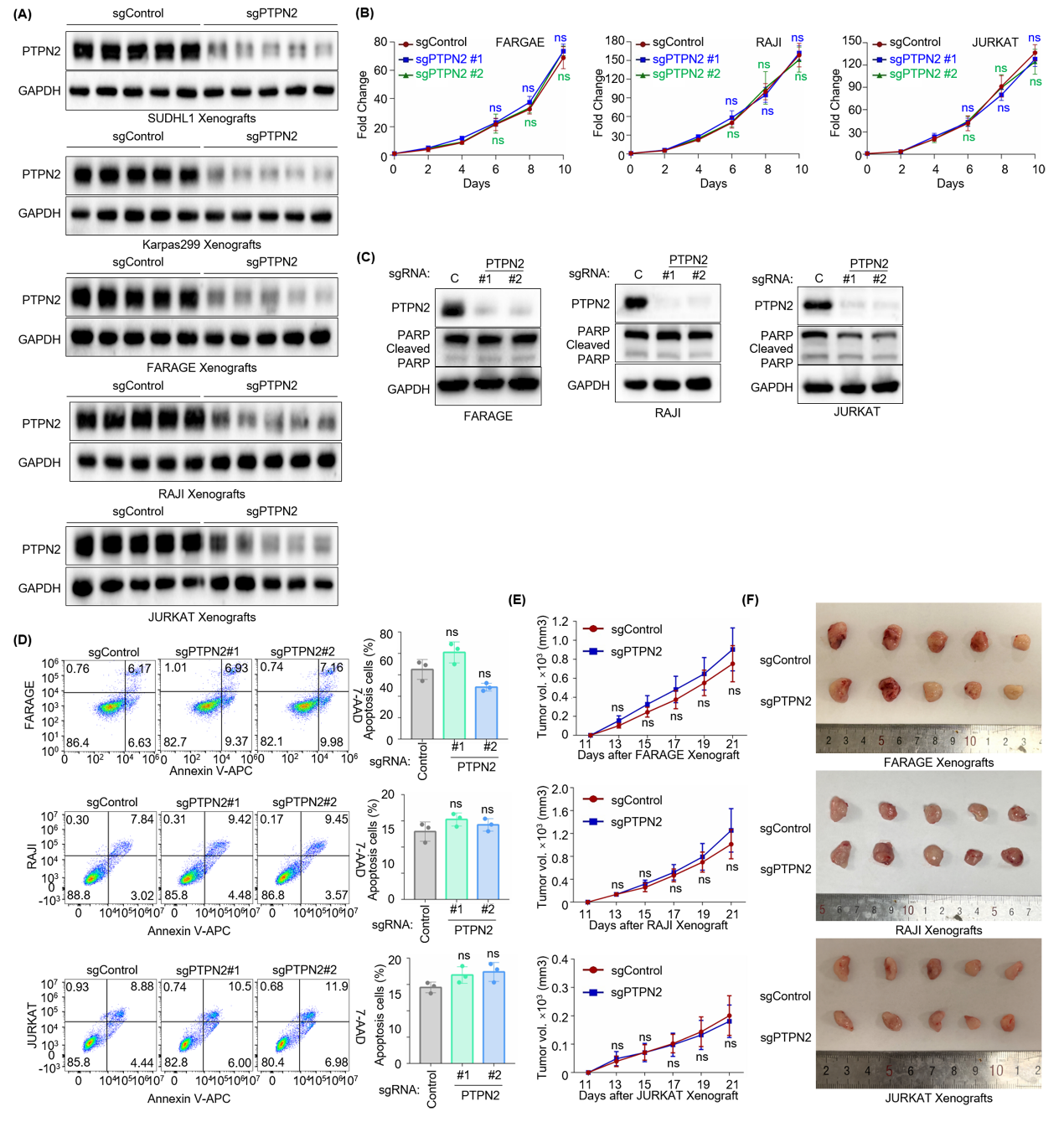
**

**Figure S1.** A) Immunoblot analysis showing PTPN2 knockout efficacy in xenograft mouse models constructed using Cas9+ SUDHL1, Karpas299, FARAGE, RAJI, and JURKAT following control or PTPN2 targeting sgRNAs expression. n = 5. B) Growth curve analysis showing the proliferation rate of Cas9+ FARAGE, RAJI, and JURKAT following control or PTPN2 targeting sgRNAs expression for 10 days. n = 3. C) Immunoblot analysis showing the expression of PTPN2, PARP, and cleaved PARP in Cas9+ FAGAGE, RAJI, and JURKAT following control (C) or PTPN2 targeting sgRNAs expression. D) Flow cytometry analysis showing cell apoptosis in Cas9+ FARAGE, RAJI, and JURKAT that express control or PTPN2 sgRNAs using annexin V-APC/7-AAD Kit. n = 3. E, F) Growth curve (E) and volume analysis (F) showing tumor proliferation and size in xenograft mouse models constructed using Cas9+ FARAGE, RAJI, and JURKAT following control or PTPN2 targeting sgRNAs expression. n = 5 per group. The data are shown as the mean ± SDs. ns, no significant. Statistical analysis in panels B, D, and E was performed by one-way ANOVA with multiple comparisons.


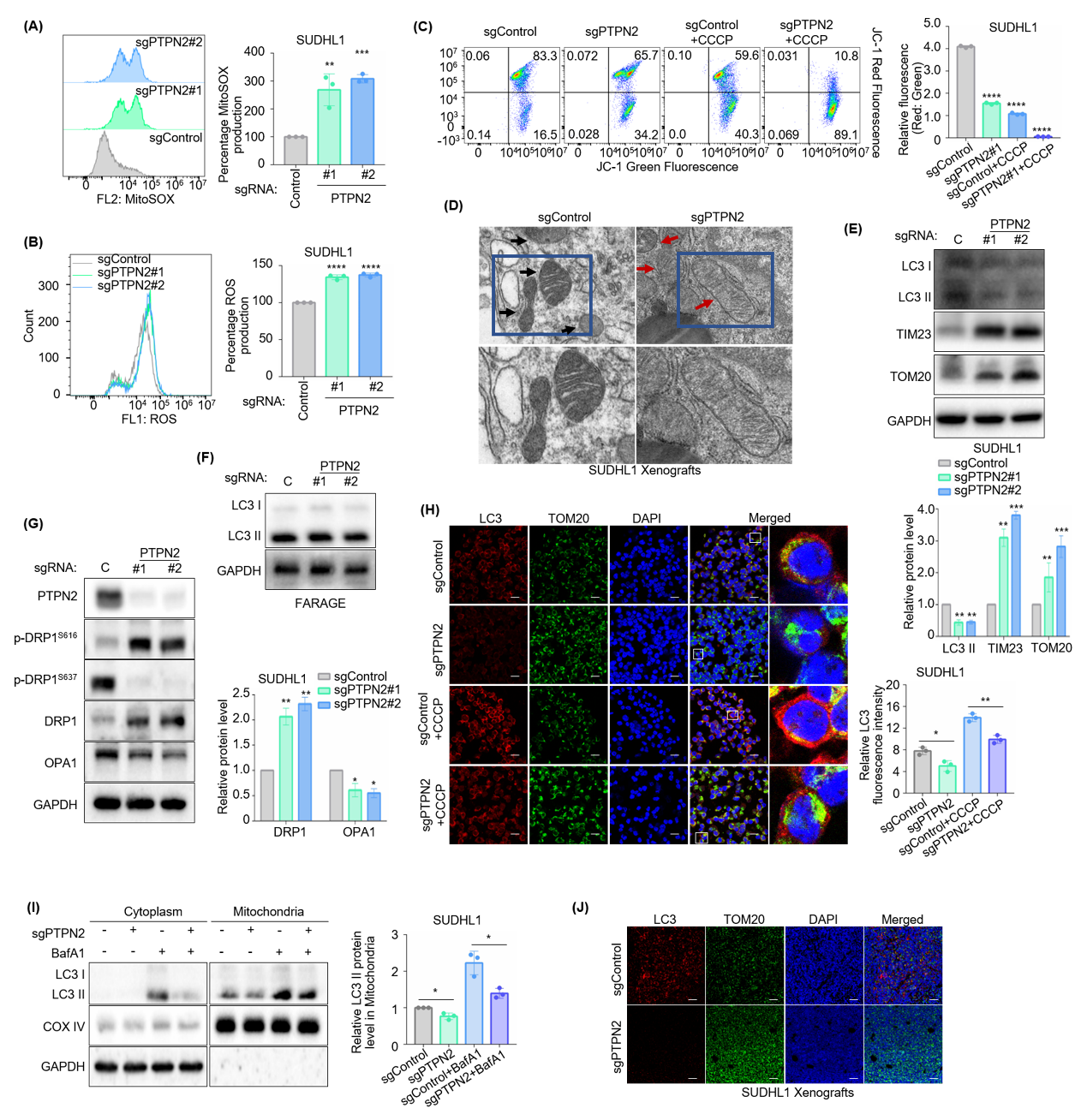


**Figure S2.** A) MitoSOX analysis in Cas9+ SUDHL1 that express control or PTPN2 sgRNAs. n = 3. B) ROS analysis in Cas9+ SUDHL1 that express control or PTPN2 sgRNAs. n = 3. C) Mitochondrial membrane potential analysis in Cas9+ SUDHL1 that express control or PTPN2 sgRNAs without or with 10 μM CCCP for 24 h by JC-1. n = 3. D) Transmission electron microscopy analysis showing mitochondrial morphology in xenograft mouse models constructed using Cas9+ SUDHL1 following control or PTPN2 targeting sgRNAs expression. x10k. n = 5. E) Immunoblot analysis showing the expression of LC3 I/II, TIM23, and TOM20 in Cas9+ SUDHL1 that express control or PTPN2 sgRNAs. n = 3. F) Immunoblot analysis showing the expression of LC3 I/II in Cas9+ FARAGE that express control or PTPN2 sgRNAs. G) Immunoblot analysis showing the expression of p‑DRP1^S616^, p‑DRP1^S637^, DRP1, and OPA1 in Cas9+ SUDHL1 that express control or PTPN2 sgRNAs. n = 3. H) Immunofluorescence analysis showing LC3 expression around mitochondria in Cas9+ SUDHL1 that express control or PTPN2 sgRNAs without or with 10 μM CCCP for 24 h. Scale bar: 20 μm. n = 3. I) Immunoblot analysis showing LC3 I/II expression in the mitochondria of Cas9+ SUDHL1 that express control or PTPN2 sgRNAs without or with 20 nM Bafilomycin A1 (BafA1) for 24 h. n = 3. J) Immunofluorescence analysis showing LC3 expression around mitochondria in xenograft mouse models constructed using Cas9+ SUDHL1 following control or PTPN2 targeting sgRNAs expression. Scale bar: 20 μm. n = 5. The data are shown as the mean ± SDs. *, *p* < 0.05; **, *p* < 0.01; ***, *p* < 0.001; ****, *p* < 0.0001. Statistical analysis in panels A-C, E, and G-J was performed by one-way ANOVA with multiple comparisons.

**
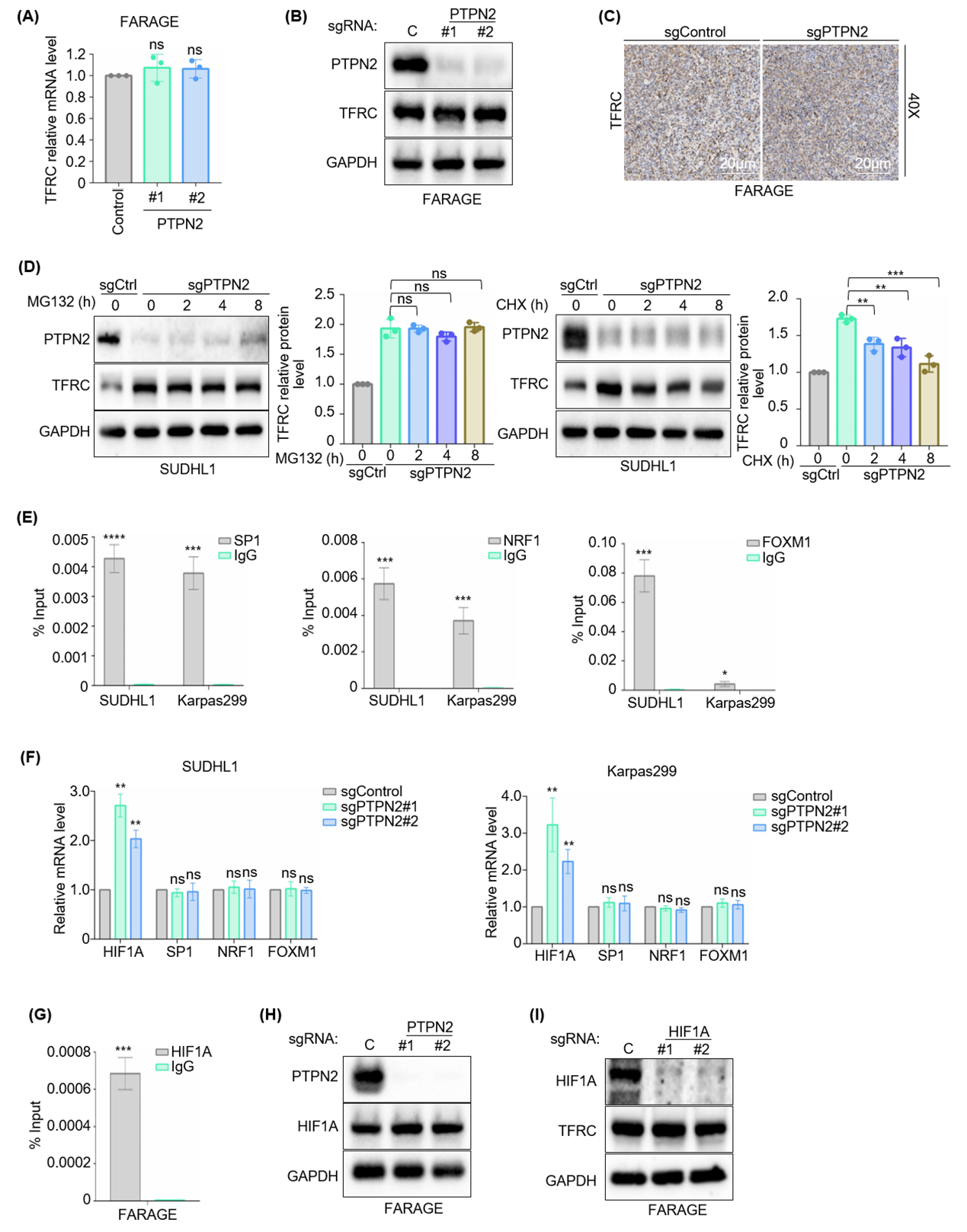
**

**Figure S3.** A, B) RT-qPCR (A) and immunoblot analysis (B) showing TFRC expression in Cas9+ FARAGE that express control (C) or PTPN2 sgRNAs. n = 3. C) Immunohistochemistry staining assay showing TFRC expression in xenograft mouse models constructed using Cas9+ FARAGE following control or PTPN2 targeting sgRNAs expression. Scale bar: 20 μm. n = 5. D) Immunoblot analysis showing TFRC expression in Cas9+ SUDHL1 that express control or PTPN2 sgRNAs with 10 μM MG132 (left) or 1 μM cycloheximide (CHX) (right) for 0, 2, 4, and 8 h, respectively. n = 3. E) ChIP-qPCR assay showing the binding between SP1, NRF1, and FOXM1 with TFRC promoter (−2000 bp to −1 bp) in SUDHL1 and Karpas299 cells. n = 3. F) RT-qPCR showing HIF1A, SP1, NRF1, and FOXM1 mRNA levels in Cas9+ SUDHL1 (left) and Karpas299 (right) that express control or PTPN2 sgRNAs. n = 3. G) ChIP-qPCR assay showing the binding of HIF1A and TFRC promoter (−2000 bp to −1 bp) in FARAGE cells. n = 3. H) Immunoblot analysis showing HIF1A expression in Cas9+ FARAGE that express control or PTPN2 sgRNAs. I) Immunoblot analysis showing TFRC expression in Cas9+ FARAGE that express control or HIF1A sgRNAs. The data are shown as the mean ± SDs. ns, no significant; *, *p* < 0.05; **, *p* < 0.01; ***, *p* < 0.001; ****, *p* < 0.0001. Statistical analysis in panels A and D-G was performed by one-way ANOVA with multiple comparisons.


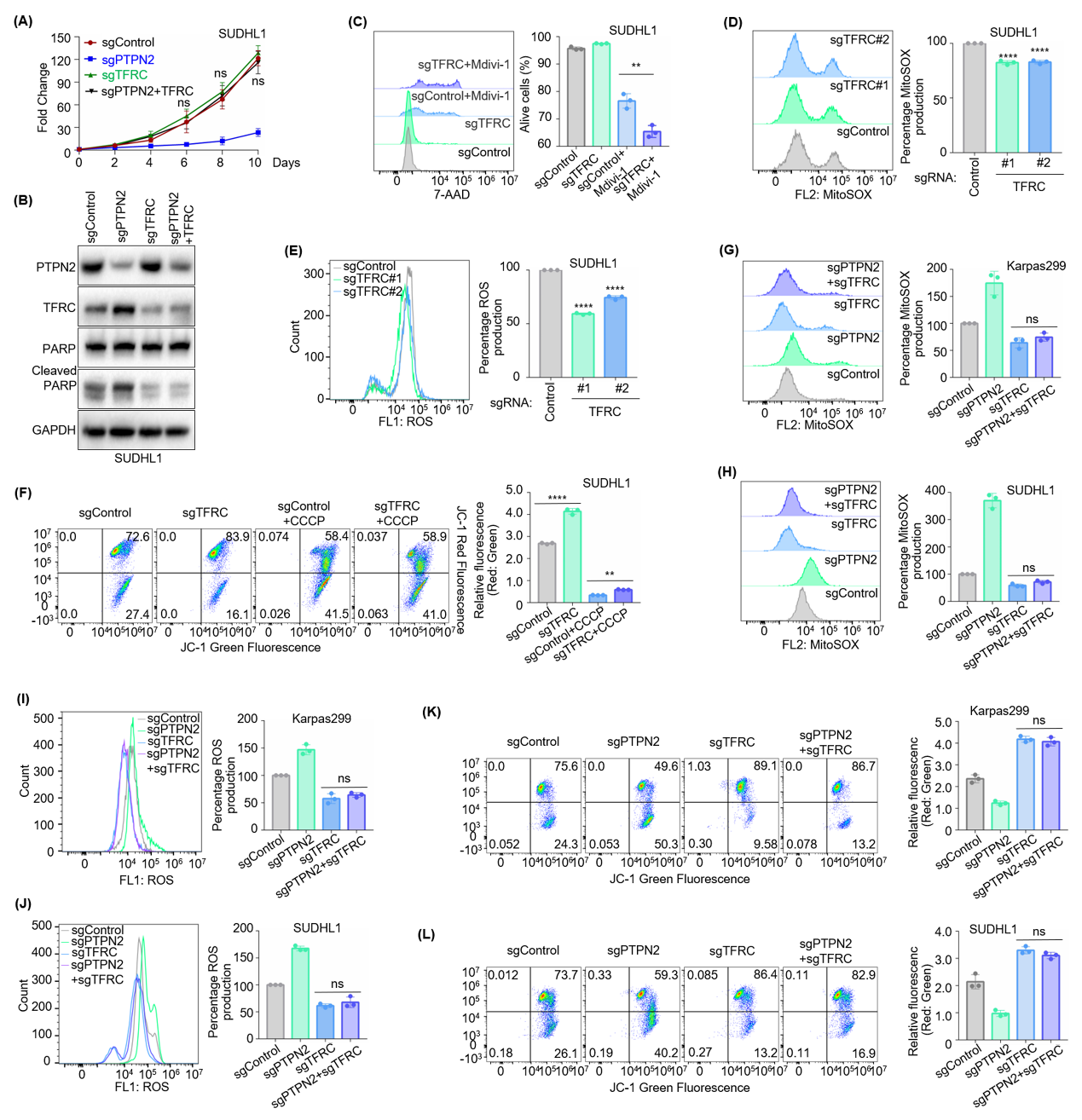


**Figure S4.** A) Growth curve analysis showing the proliferation rate of Cas9+ SUDHL1 following control, PTPN2 or TFRC targeting sgRNAs expression. n = 3. B) Immunoblot analysis showing the expression of PTPN2, TFRC, PARP, and cleaved PARP in Cas9+ SUDHL1 following control, PTPN2 or TFRC targeting sgRNAs expression. C) Flow cytometry analysis showing cell death in Cas9+ SUDHL1 that express control or TFRC sgRNAs with 10 μM mdivi-1 for 48 h using 7-AAD Kit. n = 3. D) MitoSOX analysis in Cas9+ SUDHL1 that express control or TFRC sgRNAs. n = 3. E) ROS analysis in Cas9+ SUDHL1 that express control or TFRC sgRNAs. n = 3. F) Mitochondrial membrane potential analysis in Cas9+ SUDHL1 that express control or TFRC sgRNAs without or with 10 μM CCCP for 24 h by JC-1. n = 3. G-H) MitoSOX analysis in Cas9+ Karpas299 (G) and SUDHL1 (H) that express control, PTPN2, or TFRC sgRNAs. n = 3. I-J) ROS analysis in Cas9+ Karpas299 (I) and SUDHL1 (J) that express control, PTPN2 or TFRC sgRNAs. n = 3. K-L) Mitochondrial membrane potential analysis in Cas9+ Karpas299 (K) and SUDHL1 (L) that express control, PTPN2 or TFRC sgRNAs without or with 10 μM CCCP for 24 h by JC-1. n = 3. The data are shown as the mean ± SDs. ns, no significant; **, *p* < 0.01; ****, *p* < 0.0001. Statistical analysis in panels A and C-L was performed by one-way ANOVA with multiple comparisons.


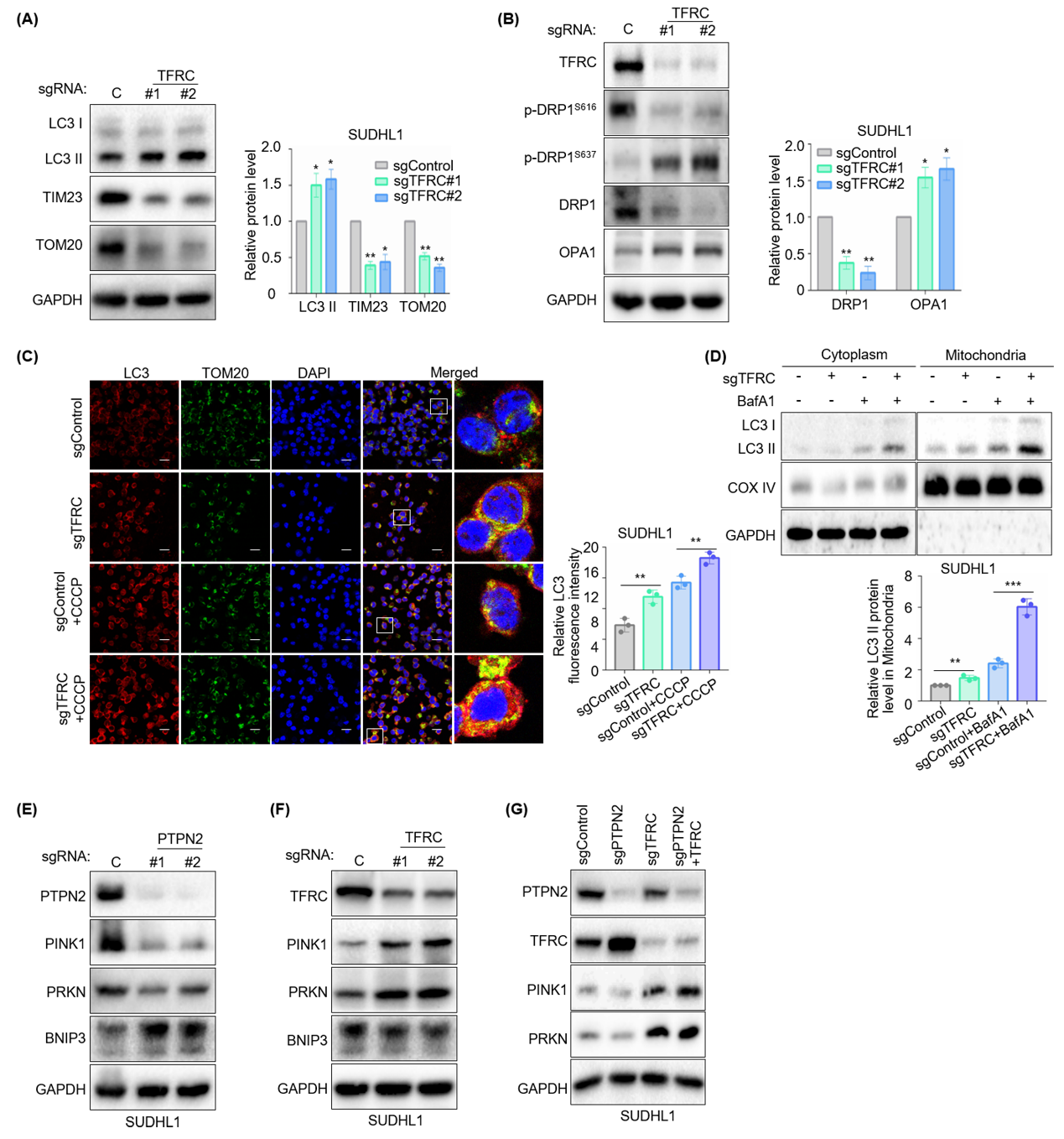


**Figure S5.** A) Immunoblot analysis showing the expression of LC3 I/II, TIM23, and TOM20 in Cas9+ SUDHL1 that express control (C) or TFRC sgRNAs. n = 3. B) Immunoblot analysis showing the expression of p‑DRP1^S616^, p‑DRP1^S637^, DRP1, and OPA1 in Cas9+ SUDHL1 that express control (C) or TFRC sgRNAs. n = 3. C) Immunofluorescence analysis showing LC3 expression around mitochondria in Cas9+ SUDHL1 that express control or TFRC sgRNAs without or with 10 μM CCCP for 24 h. Scale bar: 20 μm. n = 3. D) Immunoblot analysis showing LC3 I/II expression around mitochondria in Cas9+ SUDHL1 that express control or TFRC sgRNAs without or with 20 nM Bafilomycin A1 (BafA1) for 24 h. n = 3. E) Immunoblot analysis showing the expression of PINK1, PRKN, and BNIP3 in Cas9+ SUDHL1 that express control or PTPN2 sgRNAs. F) Immunoblot analysis showing the expression of PINK1, PRKN, and BNIP3 in Cas9+ SUDHL1 that express control or TFRC sgRNAs. G) Immunoblot analysis showing the expression of PTPN2, TFRC, PINK1, and PRKN in Cas9+ SUDHL1 following control, PTPN2 or TFRC targeting sgRNAs expression. The data are shown as the mean ± SDs. *, *p* < 0.05; **, *p* < 0.01; ***, *p* < 0.001. Statistical analysis in panels A-D was performed by one-way ANOVA with multiple comparisons.


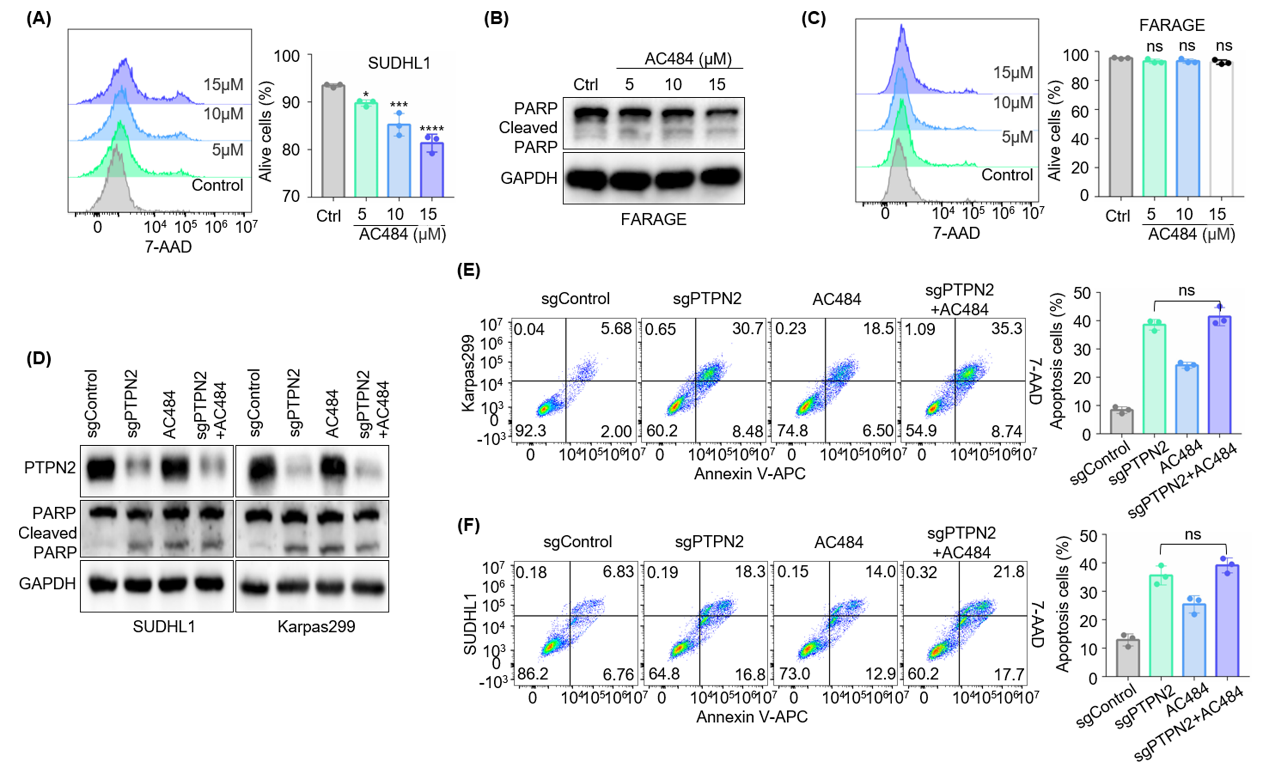


**Figure S6.** A) Flow cytometry analysis showing cell death in SUDHL1 treated with 5, 10, 15 μM AC484 for 48 h using 7-AAD Kit. n = 3. B) Immunoblot analysis showing PARP and cleaved PARP expression in FARAGE treated with 5,10,15 μM AC484 for 48h. C) Flow cytometry analysis showing cell death in FARAGE treated with 5, 10, 15 μM AC484 for 48 h using 7-AAD Kit. n = 3. D) Immunoblot analysis showing PTPN2, PARP, and cleaved PARP expression in Cas9+ SUDHL1 (left) and Karpas299 (right) treated with or without 10 μM AC484 for 48 h following control or PTPN2 targeting sgRNAs expression. E, F) Flow cytometry analysis showing cell apoptosis in Cas9+ Karpas299 (E) and SUDHL1 (F) treated with or without 10 μM AC484 for 48 h following control or PTPN2 targeting sgRNAs expression using annexin V-APC/7-AAD Kit. n = 3. The data are shown as the mean ± SDs. ns, no significant; *, *p* < 0.05; ***, *p* < 0.001; ****, *p* < 0.0001. Statistical analysis in panels A, C, E, and F was performed by one-way ANOVA with multiple comparisons.


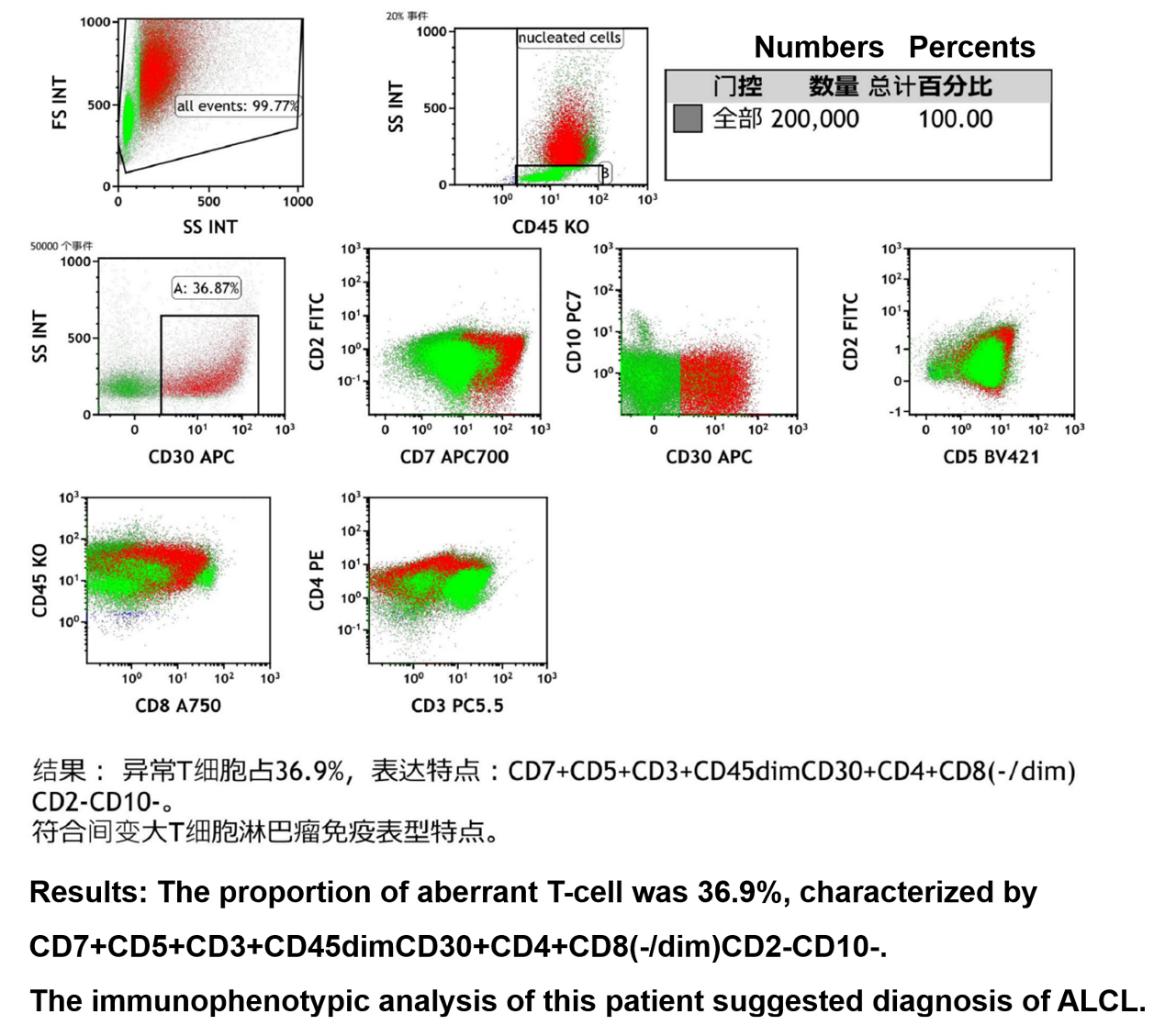


**Figure S7.** Flow cytometric results of the hydrothorax from one ALK^+^ ALCL patient. The proportion of aberrant T-cells was 36.9%, characterized by CD7+, CD5+, CD3+, CD45dim, CD30+, CD4+, CD8(-/dim), CD2-, CD10-. The immunophenotypic analysis of this patient suggested a diagnosis of ALCL.


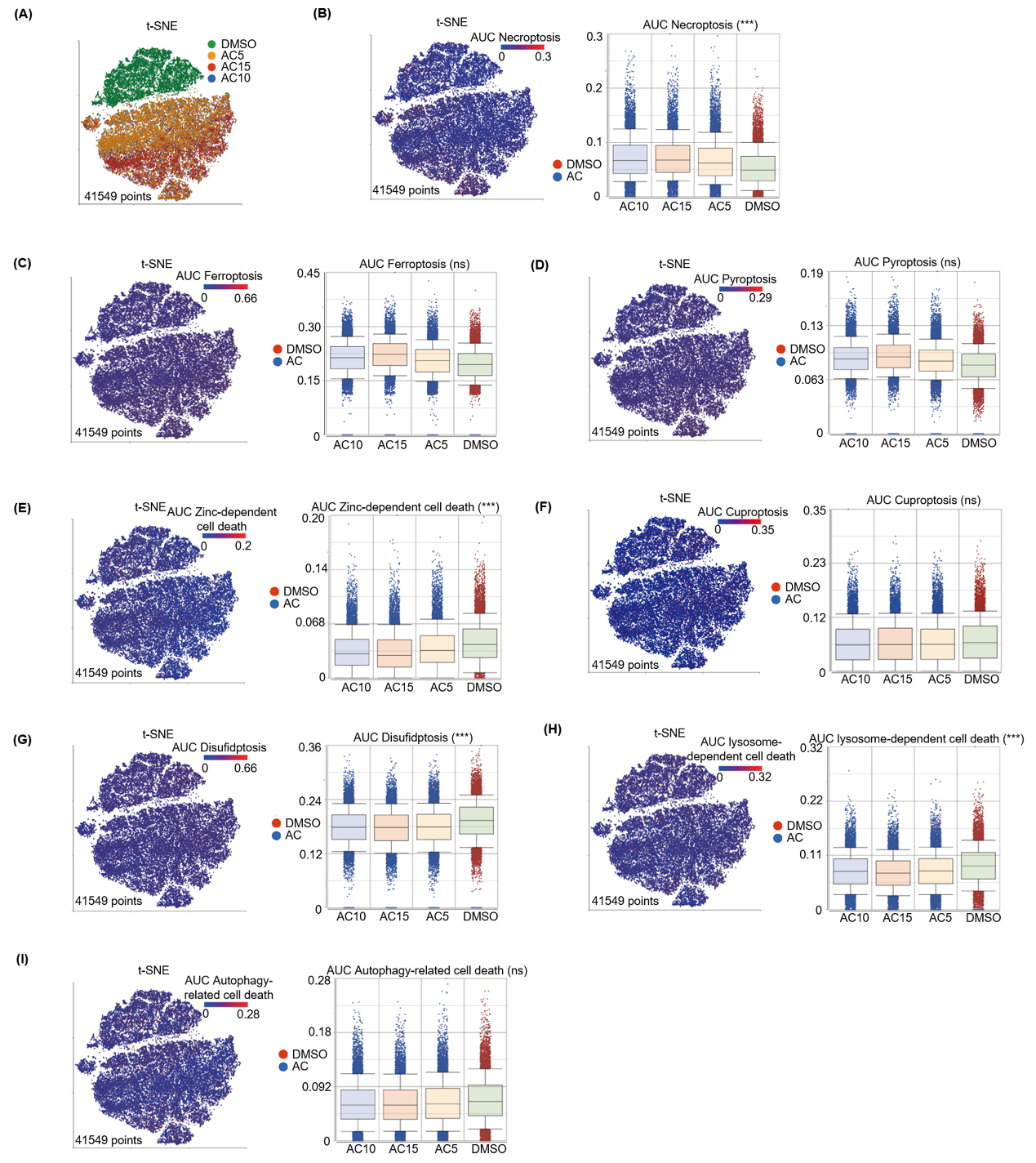


**Figure S8.** A) T-stochastic neighbor embedding (t-SNE) analysis showing the visualization of 41549 cells labeled by different concentrations of AC484 (n = 4) following quality control and unsupervised clustering in Karpas299 treated with 5, 10, 15 μM AC484 for 72 h by single-cell RNA sequencing. B-I) AUCell analysis showing the ‘Necroptosis’ activity (B), the ‘Ferroptosis’ activity (C), the ‘Pyroptosis’ activity (D), the ‘Zinc-dependent cell death’ activity (E), the ‘Cuproptosis’ activity (F), the ‘Disufidptosis’ activity (G), the ‘lysosome-dependent cell death’ activity (H), and the ‘Autophagy-related cell death’ activity (I) in Karpas299 treated with 5, 10, 15 μM AC484 for 72 h by single-cell RNA sequencing. ns, no significant; ***, *p* < 0.001. Statistical analysis in panels B-I was performed by one-way ANOVA with multiple comparisons.


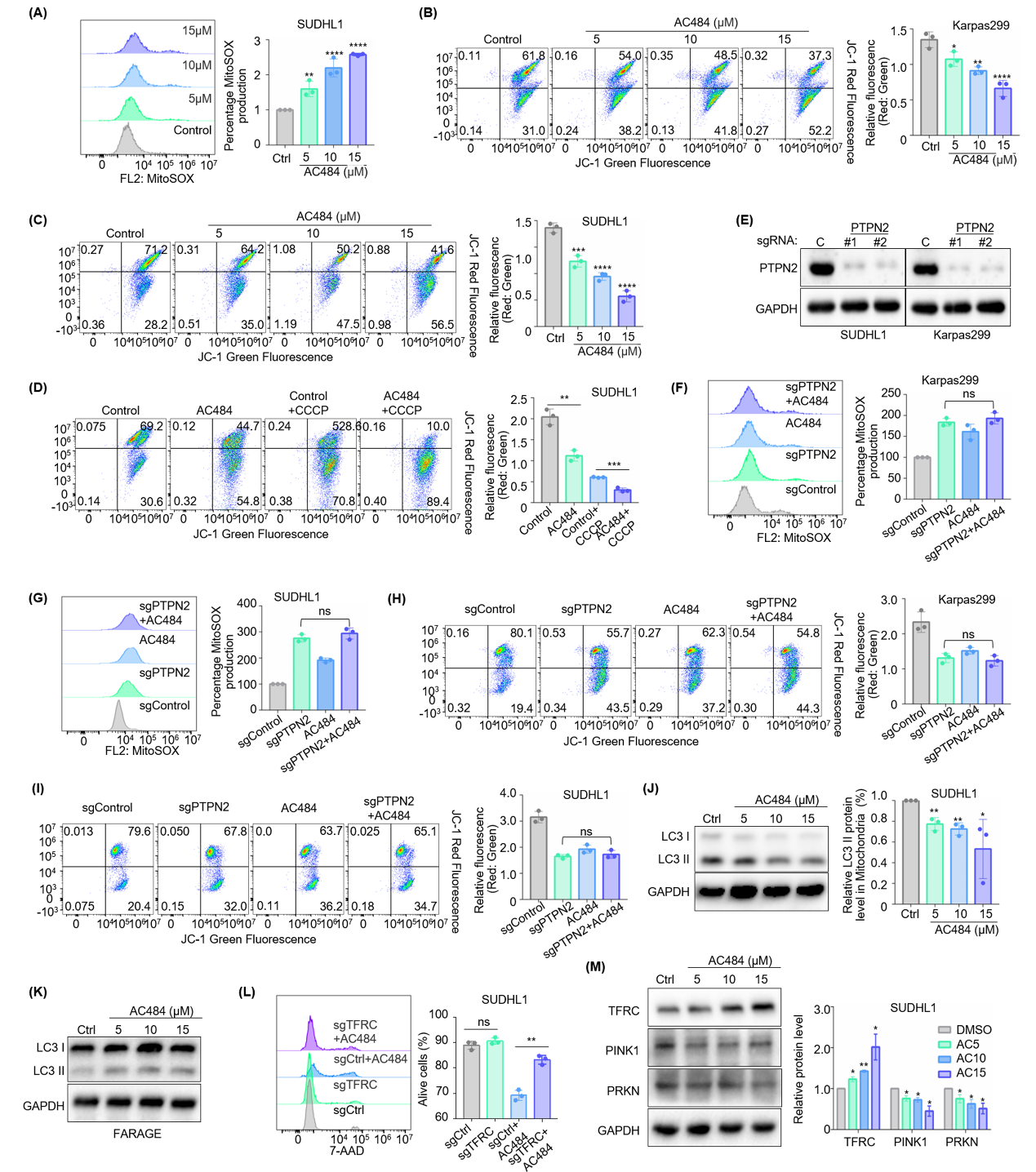


**Figure S9.** A) MitoSOX analysis in SUDHL1 treated with 5, 10, 15 μM AC484 for 48 h. n = 3. B, C) Mitochondrial membrane potential analysis in Karpas299 (B) and SUDHL1 (C) treated with 5, 10, 15 μM AC484 for 48 h. n = 3. D) Mitochondrial membrane potential analysis in SUDHL1 treated with 10 μM AC484 and/or 10μM CCCP for 24 h by JC-1. n = 3. E) Immunoblot analysis showing PTPN2 expression in Cas9+ SUDHL1 (left) and Karpas299 (right) that express control (C) or PTPN2 sgRNAs. F, G) MitoSOX analysis in Cas9+ Karpas299 (F) and SUDHL1 (G) treated with 10 μM AC484 for 48 h following control or PTPN2 targeting sgRNAs expression. n = 3. H, I) Mitochondrial membrane potential analysis in Cas9+ Karpas299 (H) and SUDHL1 (I) treated with 10 μM AC484 for 48 h following control or PTPN2 targeting sgRNAs expression. n = 3. J) Immunoblot analysis showing LC3 I/II expression in SUDHL1 treated with 5, 10, 15 μM AC484 for 48 h. n = 3. K) Immunoblot analysis showing LC3 I/II expression in FARAGE treated with 5, 10, 15 μM AC484 for 48 h. L) Flow cytometry analysis showing cell death in SUDHL1 that express control or TFRC sgRNAs with or without 10 μM AC484 and/or 10 μM mdivi-1 for 48 h using 7-AAD Kit. n = 3. M) Immunoblot analysis showing the expression of TFRC, PINK1, and PRKN in SUDHL1 treated with 5, 10, 15 μM AC484 for 48 h. n = 3. Ctrl, Control. The data are shown as the mean ± SDs. ns, no significant; *, *p* < 0.05; **, *p* < 0.01; ***, *p* < 0.001; ****, *p* < 0.0001. Statistical analysis in panels A-D, F-J, L, and M was performed by one-way ANOVA with multiple comparisons.

**Table S1.** Top 10 preferentially essential genes in ALK^+^ ALCL cell lines from Depmap

| **SUDHL1** | **Karpas299** | **KIJK** | **SUPM2** | **DEL** |
| --- | --- | --- | --- | --- |
| **PTPN2** | STAT3 | BATF3 | BATF3 | BATF3 |
| STAT3 | **PTPN2** | ZNF296 | **PTPN2** | SBNO2 |
| CWF19L1 | MYB | IKZF1 | STAT3 | EGLN3 |
| FOSL2 | IKZF1 | PACSIN2 | SUV39H1 | STAT3 |
| SCAF4 | IRF4 | **PTPN2** | SBNO2 | RORC |
| UROS | TRAPPC4 | STAT3 | PDSS1 | OR2T29 |
| ROCK2 | RORC | SP110 | API5 | **PTPN2** |
| CSTB | CCND3 | SBNO2 | HTRA2 | IRF4 |
| TIMM17A | JTB | RAB1A | PTPN1 | TRIM11 |
| VPS45 | PTPN1 | MAPKAPK2 | POU3F3 | RUNX2 |

**Table S2.** Clinical characteristics of 8 cases of ALK^+^ ALCL

|  | All patients (n=8) |
| --- | --- |
| Gender |  |
| Male | 3 |
| Female | 5 |
| Age |  |
| ≥60 years | 3 |
| <60 years | 5 |
| B symptoms |  |
| Absence | 5 |
| Presence | 3 |
| ECOG |  |
| <2 | 5 |
| ≥2 | 3 |
| Ann Arbor Stage |  |
| I-II | 3 |
| III-IV | 5 |
| Extranodal sites |  |
| 0-1 | 5 |
| ≥2 | 3 |
| LDH |  |
| normal | 7 |
| elevated | 1 |
| IPI status |  |
| 0-1 | 4 |
| 2-3 | 3 |
| 4-5 | 1 |
| BM involvement |  |
| NO | 5 |
| YES | 3 |
| PIT score |  |
| 0 | 2 |
| 1-2 | 5 |
| 3-4 | 1 |

**Table S3.** Sequences of primers and sgRNAs used for CRISPR editing

|  | Name | sgRNA sequence | Source |
| --- | --- | --- | --- |
| sgRNA | sgControl | ATTTCGCAGATCATCGACAT | Brunello Library |
|  | PTPN2 #1 SS | TGTCATGCTGAACCGCATTG | Brunello Library |
|  | PTPN2 #2 SS | AAGGAGTTACATCTTAACAC | Brunello Library |
|  | TFRC #1 SS | TCGTGAGGCTGGATCTCAAA | Brunello Library |
|  | TFRC #2 SS | TGCAGCACGTCGCTTATATT | Brunello Library |
|  | HIF1A #1 SS | AAGTGTACCCTAACTAGCCG | Brunello Library |
|  | HIF1A #2 SS | CAGTGTGGGTATAAGAAACC | Brunello Library |

**Table S4.** Primer sequences used for real-time PCR

| Name | Sense sequence (5'‒>3') | Antiense sequence (5'‒>3') |
| --- | --- | --- |
| PTPN2 | CTGCCTTTGATCATTCACCAAA | GGTCACCTGTCAGTTTTTCTTC |
| TFRC | ACCTGTCCAGACAATCTCCAG | TGTTTTCCAGTCAGAGGGACA |
| ACTIN | CCTAGAAGCATTTGCGGTGG | GAGCTACGAGCTGCCTGACG |
| HIF1A | GAACGTCGAAAAGAAAAGTCTCG | CCTTATCAAGATGCGAACTCACA |
| SP1 | TGGCAGCAGTACCAATGGC | CCAGGTAGTCCTGTCAGAACTT |
| NRF1 | AGGAACACGGAGTGACCCAA | TATGCTCGGTGTAAGTAGCCA |
| FOXM1 | CGTCGGCCACTGATTCTCAAA | GGCAGGGGATCTCTTAGGTTC |
